# Alkali Ion-Accelerated Gelation of MXene-Based Conductive Hydrogel for Flexible Sensing and Machine Learning-Assisted Recognition

**DOI:** 10.3390/gels10110720

**Published:** 2024-11-07

**Authors:** Weidan Na, Chao Xu, Lei An, Changjin Ou, Fan Gao, Guoyin Zhu, Yizhou Zhang

**Affiliations:** 1 College of Chemistry and Chemical Engineering, Xuzhou University of Technology, Xuzhou 221111, China; wdna@xzit.edu.cn; 2 Institute of Advanced Materials and Flexible Electronics (IAMFE), School of Chemistry and Materials Science, Nanjing University of Information Science and Technology, Nanjing 210044, China; X1716165558@163.com (C.X.); koma455@163.com (L.A.); gaofan@nuist.edu.cn (F.G.); gyzhu@nuist.edu.cn (G.Z.)

**Keywords:** conductive hydrogel, MXene, alkali ion, water-retention ability, machine learning

## Abstract

Conductive hydrogels are promising active materials for wearable flexible electronics, yet it is still challenging to fabricate conductive hydrogels with good environmental stability and electrical properties. In this work, a conductive MXene/LiCl/poly(sulfobetaine methacrylate) hydrogel system was successfully prepared with an impressive conductivity of 12.2 S/m. Interestingly, the synergistic effect of MXene and a lithium bond can significantly accelerate the polymerization process, forming the conductive hydrogel within 1 min. In addition, adding LiCl to the hydrogel not only significantly increases its water retention ability, but also enhances its conductivity, both of which are important for practical applications. The flexible strain sensors based on the as-prepared hydrogel have demonstrated excellent monitoring ability for human joint motion, pulse, and electromyographic signals. More importantly, based on machine learning image recognition technology, the handwritten letter recognition system displayed a high accuracy rate of 93.5%. This work demonstrates the excellent comprehensive performance of MXene-based hydrogels in health monitoring and image recognition and shows potential applications in human–machine interfaces and artificial intelligence.

## 1. Introduction

In contrast to the traditional rigid electronic products, flexible electronic devices can be easily integrated into various curved surfaces and are well-suited for applications in human–machine interfaces, health monitoring, and electronic skin, among other fields [1,2,3]. The growing popularity of flexible electronics has also further spurred a significant demand for soft conductors, which can be roughly divided into two classes based on their mechanical properties. One involves the integration of circuits onto flexible substrates or the construction of conductive networks within elastic materials [4,5]. For instance, Pang et al. prepared a highly sensitive flexible sensor capable of detecting different forms of stress such as shear and torsional forces by assembling metal-coated elastomer fibers on a polydimethylsiloxane substrate [6]. While the modulus of these electronic devices is orders of magnitude higher than that of human tissues, the mismatching of the mechanical properties and interface shedding will lead to low sensitivity and performance degradation. Another type of soft conductor is the rising conductive hydrogel. Due to their special three-dimensional hydration network structure, these hydrogels show solid properties in the macroscopic domain and liquid behavior in the microscopic domain, and their elastic modulus closely approximates that of the human tissues, making them promising candidates for biomimetic skin materials suitable for wearable technology [7,8,9]. Unfortunately, these hydrogels are susceptible to crystallization or water loss under sub-zero or elevated temperatures, resulting in device performance instability. Therefore, a series of organic solvents or salts have been introduced into conductive hydrogel systems [10,11], while the low electrical conductivity remains a limitation for their application as flexible sensors. Therefore, it is still urgent to develop new conductive hydrogels with high conductivity, outstanding performance stability, excellent biocompatibility, good tensile resilience, and self-healing characteristics.

Many 2D materials possess metallic conductivity, high carrier mobility, large specific surface area, excellent hydrophilicity, and high ionic embedment [12,13,14]. MXene as a representative 2D material displays a carrier mobility of 10^4^~10^5^ cm^2^V^−1^s^−1^, is frequently employed in constructing MXene-based conductive hydrogels for applications in biomedicine, electromagnetic interference shielding, sensing, and other fields [15,16,17,18,19,20]. For example, Zhang’s group demonstrated that strain sensors fabricated from MXene-incorporated conductive hydrogels showed outstanding stretching properties exceeding 3400% and excellent tensile strain sensitivity with a sensitivity factor of 25, which is ten times that of the original hydrogels [21]. However, the random overlap and aggregate of MXene nanosheets will lead to diminished surface area and compromised electrochemical performance. In addition, MXene hydrogels display inadequate frost resistance, rendering them unsuitable for low-temperature flexible sensing applications. Consequently, it is highly advantageous for MXene-based hydrogel flexible electronics to effectively mitigate phase separation and improve environmental stability.

Herein, MXene/LiCl/poly(sulfobetaine methacrylate) (PSBMA-LM)-based hydrogels with high stretchability (up to 1100%), electrical conductivity (up to 12.2 S/m), good adhesion, and self-healing properties were successfully prepared by incorporating MXene and LiCl into the hydrogel system. The presence of LiCl not only enhances the water retention and frost resistance of hydrogel, but also accelerates the gelation of the hydrogel via lithium bonding, which can significantly decrease the aggregate of MXene. PSBMA-LM-based flexible sensors exhibited good sensitivity across a wide strain range, enabling accurate and reliable monitoring of various human activities. Further, the flexible surface electromyographic (EMG) acquisition system can effectively collect muscle-surface electrical signals. In addition, when combined with the machine learning image recognition module, the hydrogels were endowed with intelligent sensing functions to recognize human writing activities with a high accuracy rate over 93%, and these results showed that PSBMA-LM hydrogels possess huge potential applications in flexible multifunctional electronic devices.

## 2. Results and Discussion

### 2.1. Preparation and Characterization of Conductive Hydrogels

**Figure 1:**
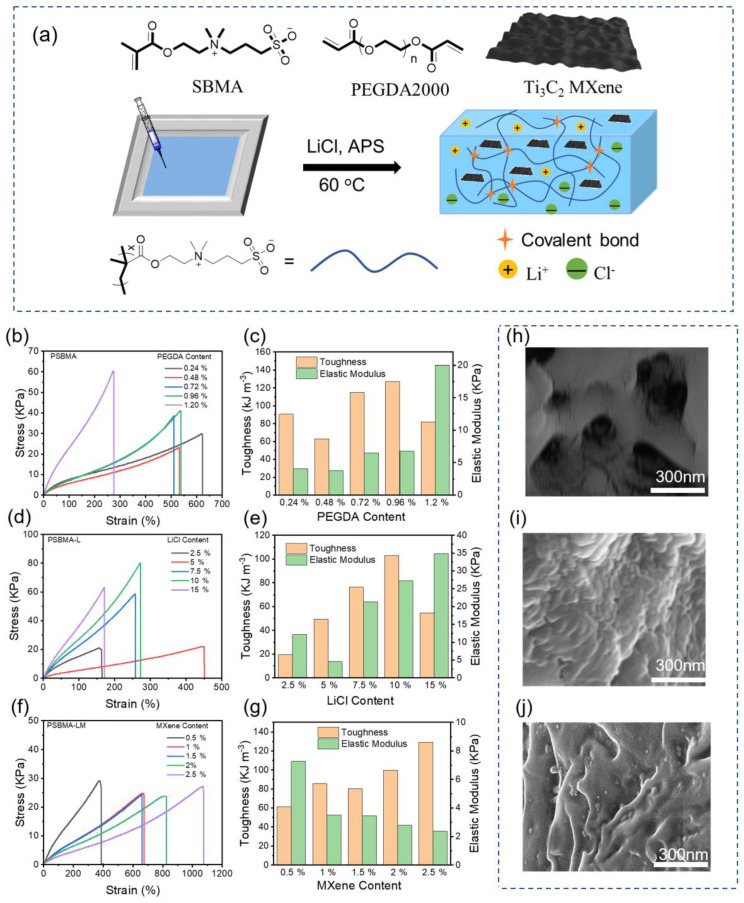
(**a**) Schematic diagram of preparation conductive PSBMA-LM hydrogel. (**b**,**c**) Tensile stress–strain curves and toughness and elastic modulus of PSBMA hydrogel with different PEGDA contents. (**d**,**e**) Tensile stress–strain curves and toughness and elastic modulus of PSBMA-L hydrogel with different LiCl contents. (**f**,**g**) Tensile stress–strain curves and toughness and elastic modulus of PSBMA-LM hydrogel with different MXene contents. SEM image of freeze-dried PSBMA hydrogel (**h**), PSBMA-L hydrogel (**i**), and PSBMA-LM hydrogel (**j**).

Since zwitterion polymers contain a large number of anions and cations and can interact with MXene via electrostatic interaction and hydrogen bonding, they would mitigate phase separation between MXene and hydrogel. To further enhance conductivity and water retention ability, LiCl was added into the hydrogel. As indicated in Figure 1a, the conductive hydrogel based on a poly-zwitterion framework was prepared via free radical polymerization of sulfobetaine methacrylate (SBMA). It is well known that good mechanical elasticity is an indispensable characteristic of flexible conductive hydrogel sensors. We investigated the effects of varying amounts of crosslinking agent (PEGDA), LiCl, and MXene on the mechanical properties of hydrogels to optimize the additive ratios (Figure 1b–g). In our experiment, the quantities of the crosslinking agent, LiCl, and MXene were determined based on the mass of the monomer. As indicated in Figure 1b, increasing PEGDA content from 0.2% to 1.2% resulted in a rising trend in tensile strength for the PSBMA hydrogel, while its elongation at break decreased from 622% to 287%. When the PEGDA content was 1.2%, the hydrogel exhibited a high degree of crosslinking with a tensile strength exceeding 60 Kpa, but the elongation at break was relatively small. Therefore, the higher content of the crosslinking agent PEGDA was not suitable for applications in flexible devices. The toughness and elastic modulus reflect the mechanical properties of the hydrogel, and the corresponding changes in toughness and Young’s modulus are shown in Figure 1c. When the PEGDA content was 0.72%, the hydrogel had good toughness and elastic modulus as well as appropriate flexibility, so this content was chosen for further study. Secondly, we prepared PSBMA-L hydrogels containing 0.72% of PEGDA with various LiCl contents to evaluate their tensile properties. As shown in Figure 1d, compared to PSBMA hydrogel without LiCl addition, incorporating LiCl generally reduced the elongation at break of the hydrogel, and the tensile strength of PSBMA-L hydrogel was compensated with a gradual increase in LiCl content. With the increase in LiCl content, the toughness of PSBMA-L hydrogel gradually increased until reaching a peak value at approximately 10% LiCl (Figure 1e), and then decreased, which could be attributed to the charged free ions weakening hydrogen bonding to a certain extent through the interaction between charges. When the LiCl content reached 15%, the mechanical properties of the hydrogel showed a downward trend, further confirming that 10% was the optimal dosage of LiCl. Overall, we chose PSBMA-L hydrogel containing 10% LiCl for subsequent research.

Finally, PSBMA-LM hydrogels containing 0.72% PEGDA and 10% LiCl with varying MXene contents were prepared and their tensile properties were measured. MXene nanosheets were synthesized by etching aluminum from MAX phase with an in situ HF-forming etching agent. It was clearly shown that MXene had a layered microstructure via X-ray diffraction (XRD) and scanning electron microscopy (SEM) (Figures S1 and S2). Since the incorporation of MXene would destroy the network structure of the PSBMA-L hydrogel to a certain extent, making the connection between molecular chains less firm, and reducing the cross-linking density, adding MXene generally increased in the elongation at break for PSBMA-LM hydrogel (Figure 1f). Although the Young’s modulus gradually decreased with increasing MXene content, the toughness was enhanced (Figure 1g). Therefore, 1% MXene content compromised the mechanical performance of PSBMA-LM hydrogel, while the toughness still exceeded 80 KJ m⁻^3^. To meet the needs of flexible sensing, we ultimately selected a PSBMA-LM hydrogel containing 1% MXene content for the following experiments. Above all, the optimal contents of PEGDA, LiCl, and MXene in PSBMA-LM hydrogels were determined to be 0.72%, 10%, and 1%, respectively.

We further investigated the effect of additives on the microstructure of hydrogel by SEM. As illustrated in Figure 1h, due to insufficient freezing resistance, a large number of pores were observed in PSBMA hydrogel. After adding LiCl, PSBMA-L hydrogel exhibited improved freeze resistance, and the pore size was further reduced owing to the salting out effect (Figure 1i). Thanks to the layered structure of MXene, the hydrogel could be randomly distributed between the layered voids of MXene, so the overall microstructure of PSBMA-LM hydrogel was ridge-like (Figure 1j). The well-dispersed MXene in PSBMA-LM hydrogel will facilitate superior stretchability and enhance response behavior.

### 2.2. Mechanism of Accelerating Gelation

**Figure 2:**
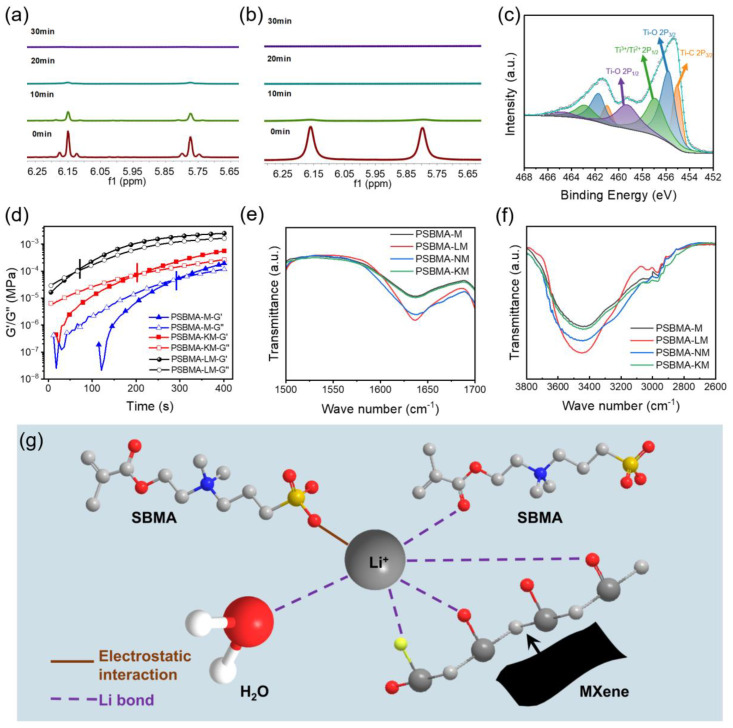
(**a**) ^1^H NMR spectra of PSBMA hydrogel. (**b**) ^1^H NMR spectra of PSBMA-L hydrogel. (**c**) XPS spectra of Ti 2p spectra of MXene. (**d**) Rheological behavior test of PSBMA, PSBMA-L, and PSBMA-LM hydrogels. (**e**,**f**) FTIR spectra of freeze-dried PSBMA-M, PSBMA-LM, PSBMA-NM, and PSBMA-KM hydrogels. (**g**) Schematic of the lithium bond in PSBMA-LM hydrogel, the colored balls represent different atoms (red: oxygen atom; grey: carbon atom; blue: nitrogen atom; white: hydrogen atom).

During the preparation processes for PSBMA-L hydrogels, we noted that the precursor solution had a tendency towards spontaneous gelation without thermal-triggering radical polymerization. Nuclear magnetic resonance (NMR) was employed to monitor the polymerization reaction over time. As shown in Figure 2a,b, the chemical shifts corresponding to vinyl hydrogens of the monomer appeared at ~6.15 and 5.77 ppm, and the NMR signals diminished significantly and nearly disappeared within ten minutes in the PSBMA-L system, indicating that most SBMA monomers were converted into oligomers and polymers, and that LiCl could accelerate the polymerization rate. Subsequently, we conducted four controlled trials to directly visualize the gelation process of PSBMA, PSBMA-L, PSBMA-M, and PSBMA-LM (Figure S3). Within just 60 s, the PSBMA-LM hydrogel has been completely gelated, while the PSBMA-M and PSBMA-L hydrogels were in a semi-gel and viscous state, respectively. The experimental results evidenced that both MXene and LiCl could collectively accelerate the polymerization of SBMA. As shown in Figure 2c, the XPS spectrum of Ti 2p binding energies at 455.08, 455.78, 456.98, and 459.38 eV corresponded to Ti–C, Ti–O, Ti^2+^/Ti^3+^, and Ti–C, respectively, showing that MXene contained a large number of low-valence Ti species, which can endow MXene with good reducibility and produce radicals by redox reactions with peroxide initiator APS [22]. However, MXene alone produces a limited number of free radicals, which is the root of the semi-gelation state observed in PSBMA-M. Impressively, the synergistic effect of MXene and LiCl in PSBMA-LM is favorable to speed up the gelation process and form a conductive hydrogel within one minute.

To further verify this hypothesis, other alkali chlorides including NaCl and KCl were added. PSBMA-NM and PSBMA-KM refer to PSBMA-NaCl-MXene and PSBMA-KCl-MXene hydrogels, respectively. As shown in Figure S4, both PSBMA-LM and PSBMA-NM hydrogels fully became gels within 60 s, while PSBMA-KM hydrogel was not completely gelated and PSBMA-M was still a sticky solution. In comparison with PSBMA-M, the addition of alkali chloride obviously promoted the gelation processes, but the gelation promoting ability of Li^+^ and Na^+^ was much greater than that of K^+^. Furthermore, rheological tests of PSBMA-M, PSBMA-LM, and PSBMA-KM hydrogels were also carried out to monitor the gelation process. When the energy storage modulus (G′) is equal to the loss modulus (G″), this represents the gelation point of the system. As shown in Figure 2d, the gelation time points of PSBMA-M, PSBMA-LM, and PSBMA-KM were 291.26, 66.8, and 200.6 s, respectively. Compared with PSBMA-M and PSBMA-KM hydrogels, the addition of Li^+^ dramatically accelerates the gelation of the hydrogel. In addition, the gelation rate of PSBMA-LM hydrogel was about 3 times faster than that of the PSBMA-KM hydrogel, which once again verified that the gelation promotion ability of Li^+^ was higher than that of K^+^.

To reveal the role of alkali ions in gelation, the hydrogels were further characterized by infrared spectroscopy. The stretching and bending vibrational modes of H_2_O appear at ~3450 and 1635 cm^−1^, respectively, which are sensitive to the surroundings. As shown in Figure 2e,f, compared with PSBMA-M, PSBMA-KM hydrogel had similar vibrational intensity, while the peak strength of freeze-dried PSBMA-LM and PSBMA-NM hydrogels was significantly stronger, suggesting that Li/Na bonds may form between cations and water molecules [23,24,25]. In addition, Li^+^ or Na^+^ easily binds to SBMA’s negatively charged ${\mathrm{SO}}_{3}^{-}$ through electrostatic interaction (Figure 2g). Moreover, there are a large number of H_2_O molecules and C=O bonds in the polymer SBMA, and the Li^+^ (Na^+^) can form a lithium (sodium) bond with both C=O and H_2_O, which has been confirmed in previous reports [26]. Additionally, a lithium bond is stronger than a sodium bond, so gelation in the presence of Li^+^ happens more rapidly rather than that with Na^+^. As a consequence, all the interactions jointly speed up the gel formation, and the conductive hydrogel will be prepared within one minute.

### 2.3. Mechanical and Electrical Properties

**Figure 3:**
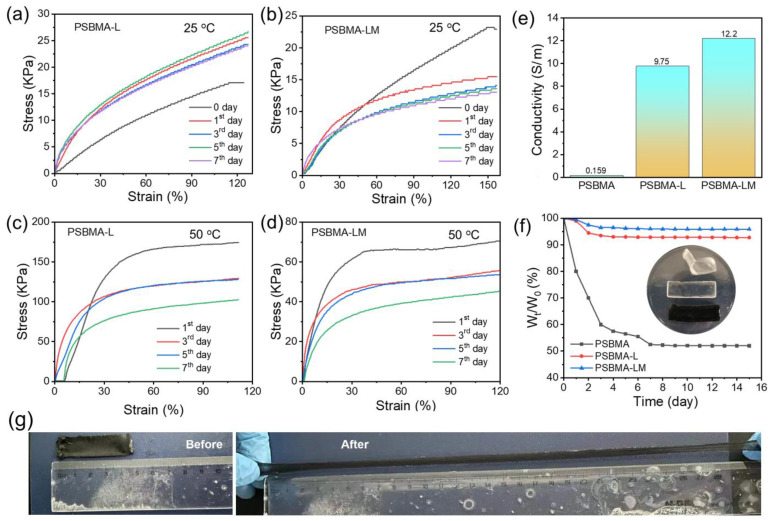
(**a**,**b**) The stress–strain curves of PSBMA-L and PSBMA-LM hydrogels within seven days at 25 °C. (**c**,**d**) The stress–strain curves of PSBMA-L and PSBMA-LM hydrogels within seven days at 50 °C. (**e**) Conductivity of PSBMA, PSBMA-L, and PSBMA-LM hydrogels. (**f**) Weight ratio of PSBMA, PSBMA-L, and PSBMA-LM hydrogels at the natural environment for different periods. Inset: Images of the as-prepared hydrogels. (**g**) Images of PSBMA-LM hydrogel before and after stretching at −20 °C.

In order to better evaluate the service life of the hydrogel-based flexible sensors, PSBMA-L and PSBMA-LM hydrogels were placed at 25 °C for 7 days, and their mechanical properties were tested under consistent strain conditions. As shown in Figure 3a,b, PSBMA-L and PSBMA-LM hydrogels maintained good mechanical properties over the course of 7 days. Similarly, we evaluated the mechanical properties of the hydrogels by placing both the samples at 50 °C for 7 days. A significant increase in tensile strength was observed in both hydrogels related to the loss of water at elevated temperature, respectively, but the hydrogels still maintained good flexibility (Figure 3c,d). In addition, the cyclic stress–strain curves of PSBMA-LM hydrogel under different strains (15%, 25%, 40%, 50%, 65%, and 80%) were tested according to the method in Video S1. It could be seen that PSBMA-LM hydrogel had a good recycling rate and can be used as a flexible sensing device for human monitoring (Figure S5). Collectively, the PSBMA-LM hydrogel shows robust mechanical properties, which can effectively meet the requirements to serve as flexible sensors.

Given the high hygroscopic property of LiCl (Figure S6), the dehydrated PSBMA-L and PSBMA-LM hydrogels could effectively absorb moisture from the surrounding environment and achieve self-regeneration. The water content of PSBMA-L and PSBMA-LM hydrogels could be restored to about 90% after placing them at 25 °C and 54% relative humidity for 4 h, while the PSBMA hydrogel did not have any regeneration ability (Figure S7). As shown in Figure 3f, PSBMA-L and PSBMA-LM hydrogels still retained over 90% water content after 15 days, showing good water retention ability. Interestingly, the PSBMA-LM hydrogel displayed good anti-freezing performance and still maintained good tensile properties at −20 °C (Figure 3g).

Electrical conductivity (σ) plays a key role in the sensitivity and response rate of flexible sensors. As shown in Figure 3e, the σ of PSBMA hydrogel was 0.159 S/m, while PSBMA-L hydrogel showed a high σ of 9.75 S/m for the ionic conduction of LiCl. Furthermore, due to the synergistic ionic and electronic conduction behaviors of LiCl and MXene, the σ of PSBMA-LM hydrogel further increased to 12.2 S/m, positioning it among the highest values reported (Table S1). That means the introduction of MXene could not only improve the σ but also enhance the flexibility of as-prepared hydrogels. In addition, conventional flexible sensors usually require some kind of adhesive to make them adhere to the surface of the skin when used. Interestingly, the PSBMA-LM hydrogel displayed good adhesion capacity, and perfectly adhered to various materials and human skin without additional adhesives (Figure S8). We further evaluated the self-healing properties of the PSBMA-LM hydrogel, and the mechanical properties of the hydrogel were restored to a certain extent after 10 min of self-healing (Figure S9), which could be attributed to the electrostatic interaction and dynamic hydrogen bonding in the hydrogel system [10,27].

### 2.4. Hydrogel-Based Flexible Sensing Performance

**Figure 4:**
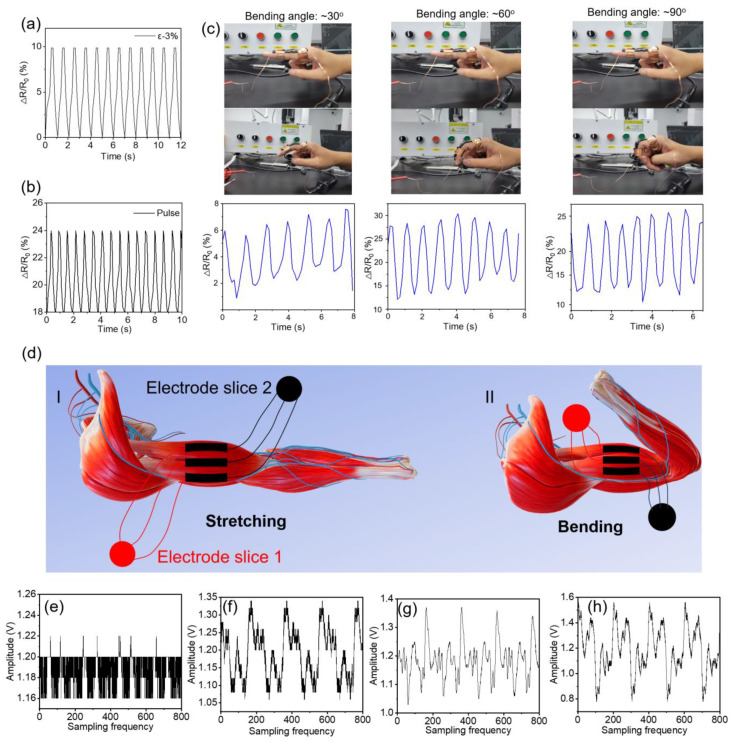
(**a**) Relative resistance changes in PSBMA-LM hydrogel-based sensor under a strain of 3%. (**b**) Real-time monitoring of pulse based on PSBMA-LM sensors. (**c**) Photographs of PSBMA-LM hydrogel sensor attached to finger for monitoring the bending motion and the relative resistance change in the sensor with different bending angles. (**d**) The schematic diagram of monitoring of electromyographic signals during arm movement by flexible sensing system. (**e**) Characteristic amplitude changes when the human body is not attached. (**f**) Characteristic amplitude changes when connected to human muscle surface. (**g**) Characteristic amplitude changes during arm muscle centrifugal movement. (**h**) Characteristic amplitude changes during knee movement.

Inspired by the excellent stability, high conductivity, and flexibility of the PSBMA-LM hydrogel, we further prepared a series of hydrogel-based flexible sensors. First, we monitored the sensor’s response to small strains. When the cyclic strain was ~3%, 6%, and 9%, all the sensors displayed excellent response ability (Figure 4a and Figure S10). In addition, the sensors showed good response ability to large strains (Figure S10). To assess the strain sensitivity of the PSBMA-LM-based sensor, a gauge factor was calculated from the change in the relative resistance with the strain. As illustrated in Figure S11, the gauge factor was 0.6 and 1.3 in the strain range of 0–220% and 220–600%, respectively. The results evidenced that the change in relative resistance was linearly correlated with the appropriate strain. Furthermore, we conducted long-term monitoring of sensing performance under 100% strain (Figure S12). It can be seen that the sensor displayed good sensing stability and had potential application in flexible electronics and intelligent detection, so we further used the sensor for real-time motion detection. As shown in Figure 4c, as the degree of finger bending increased from ~30° to ~90°, the change in the relative resistance of the hydrogel showed a stable and clear signal. The larger the bending angle, the stronger the output signal. In addition, the sensor could accurately detect a series of movement behaviors with small joint deformation or large joint movement such as elbow, wrist, knees, and heartbeat (Figure 4b and Figure S12).

Presently, the motion detection of the human body is mainly limited to the change in resistance, and there are no obvious electrical signal characteristics to accurately distinguish between types of movements. In addition, the amplitudes of human surface EMG signals and pulse signals are small and susceptible to interference. In order to solve these problems and reduce the impact of electrical signal drift on the sensor, we designed a flexible surface EMG acquisition and processing system with a simple structure and low cost based on the communication between the upper computer of LabVIEW and STM32 (Figure S13). The system’s bandpass filter, 50 Hz notch, and right leg drive circuit greatly suppressed noise interference and improved the signal-to-noise ratio. Then, the processed signal was digitally filtered and rectified to extract the features of the EMG signals. Compared with the resistance change rate of motion joint detection, EMG could provide more detailed information and a better solution for human health monitoring [28,29]. To test the responsive ability of EMG signals, we designed a PSBMA-LM hydrogel sensor, which was connected to the signal detector through the electrode sheet at both ends and detected the changes in EMG signals during movement (Figure 4d). To eliminate the interference of environmental background, amplitude change was first detected in the air. The overall amplitude tended to be stable, and there was signal fluctuation interference occasionally, which was caused by air vibration (Figure 4e). When the sensor was attached to the human body without any action, its amplitude changes (Figure 4f) were obviously different from those placed in the natural environment. We could observe the EMG characteristics in the natural state and mark them with the blue dashed-line box in the figure. When we performed repeated muscle centrifugation exercises, the EMG changed again (Figure 4g), and the signal mode had fine structures and was different from the background. In addition, we also detected the EMG in the knee area and other human body parts, which also obtained unique characteristic signals (Figure 4h and Figure S14). Therefore, we have turned a single change in resistance when different parts of the body move into a more pronounced characteristic signal change. This also lays a foundation for the following research on human–computer interaction systems.

### 2.5. Machine Learning

**Figure 5:**
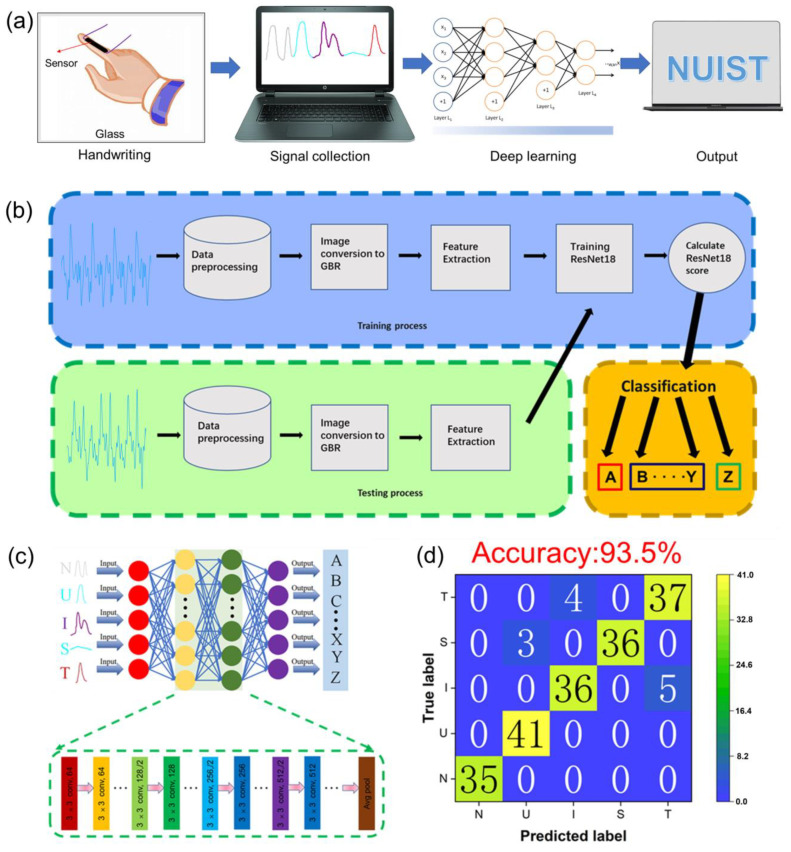
(**a**) The working principle behind the handwriting letter recognition system based on a PSBMA-LM hydrogel sensor. (**b**) Schematic diagram of the machine learning image recognition process based on the ResNet18 model. (**c**) Detailed framework of the ResNet18 model. (**d**) Confusion matrix of image recognition results based on ResNet18.

Consequently, a handwriting letter recognition system was designed based on machine learning image recognition technology. After wearing a sensor on the finger, 26 capital letters were written in the air with the finger (Figure 5a). Since each letter has its own corresponding writing and force modes, there will be 26 different characteristic electrical signals (Figure S15). We constructed a model based on deep learning to classify and recognize the characteristic electrical signals of different letters. A large number of characteristic electrical signals for 26 capital letters were collected, and then the image data were processed. The whole modeling process is shown in Figure 5b. Firstly, the image was transformed into GBR mode after feature extraction, and the model was trained. Different algorithms had a non-negligible impact on the accuracy of the model, and we evaluated the preliminary accuracy of different algorithms (Table S2). Figures S16–S21 showed the confusion matrices of different identification results. Then, the trained results would be classified and identified. After the model was established, during forecasting, when an electrical signal was input to the model, it would output a result after feature extraction and recognition.

Machine learning is considered to be an effective method to improve recognition accuracy by extracting more hidden features from seemingly unrelated data sets, which iterative learning and training of large samples can effectively identify and accurately predict [30]. The model used here was the ResNet18 model, where we took data on different people writing different letters and created a complete data set (1002), dividing the training set and the test set in an 8:2 ratio. As a classical deep convolutional neural network, the basic structure of the ResNet18 model includes an input layer (accepting GBR images) [31], convolutional layers (4 convolutional layers in total, each of which uses a 3x3 convolutional kernel and ReLU activation function to extract local features of images), and residual blocks (8 residual blocks in total). Each residual block consists of two convolutional layers and one jump connection, which is used to solve the gradient disappearance and gradient explosion problems of deep convolutional neural networks, global average pooling layer (global average pooling of feature graphs to transform feature graphs into one-dimensional vectors), fully connected layer (including a fully connected layer with a size of 1000), and, for categorizing output, an output layer (using softmax activation function to generate a probability distribution of 1000 categories). For example, we selected 5 target handwritten letters (“N”, “U”, “I”, “S”, “T”) for testing. In order to improve the recognition accuracy, more than 30 samples were taken for each target electrical signal image for complex high-dimensional classification, and each hidden layer took the output of the previous layer as its input (Figure 5c). The results showed that the recognition accuracy of 5 different character signals reached 93.5% (Figure 5d). Therefore, combining flexible sensors with machine learning pattern recognition, which could accurately achieve signal conversion functions, shows great potential in the field of artificial intelligence.

## 3. Conclusions

In summary, a multifunctional conductive hydrogel, PSBMA-LM, was prepared via Li^+^-promoting gelation of an MXene-based hydrogel. The incorporation of LiCl can not only improve the water retention ability of the hydrogel, but also accelerate the gelation of the hydrogel by lithium bonding interactions. The as-prepared PSBMA-LM hydrogel possesses remarkable flexibility, a high electrical conductivity of 12.2 S/m, and outstanding self-healing and adhesive performance. Flexible sensors based on PSBMA-LM hydrogels showed good sensitivity in detecting subtle human movements. In addition, the flexible surface EMG acquisition system could successfully collect muscle surface electrical signals and is expected to extend to clinical applications such as motor nerve stimulation and vagus nerve stimulation for the treatment of various intractable diseases, which is conducive to the development of the next generation of bioelectronics. Finally, an efficient handwriting letter recognition system was developed by the combination of flexible sensing of the PSBMA-LM hydrogel with a deep learning image recognition model, revealing its significant potential for wearable electronics and artificial skin applications.

## 4. Materials and Methods

Materials: 3-[N,N-dimethyl-[2-(2-methylpropyl-2-enyloxy)ethyl]ammo-nium]propane-1-sulfonate endosulfate (SBMA) was purchased from Anhui Zesheng Technology Co., Ltd. (Anhui Province, China) Poly(ethylene glycol) diacrylate (PEGDA_2000_), LiCl, NaCl, and KCl were purchased from Aladdin Biochemical Technology Co. Ltd. (Shanghai, China) (NH₄)₂S₂O_8_(Ammonium persulphate, APS) was purchased from Wokai Biotechnology Co., Ltd. (Beijing, China)

Preparation of MXene nanosheets: MXene nanosheets (Ti_3_C_2_T_x_) were synthesized by etching aluminum from the MAX phase using an in situ HF-forming etchant. The etchant was prepared by dissolving LiF (1.2 g) in 9 M HCl (20 mL). Subsequently, Ti_3_AlC_2_ (1.0 g) was added to the etchant and continuously stirred for 24 h at 42 °C. The resulting etching suspension was washed with deionized (DI) water through centrifugation at 5000 rpm (5 min per cycle) several times, until the pH of the supernatant reached approximately 6. The centrifuged multilayer MXene nanosheets were collected and freeze-dried for 24 h.

Preparation of the hydrogel: The hydrogels were prepared by initiating radical polymerization at 60 °C. The hydrogels with different contents of PEGDA, LiCl, and MXene were obtained by the following method. First, SBMA monomer (1.0 g) and APS (0.020 g) were added into 2 mL distilled water. In order to dissolve the reagents fully, a beaker containing the reagents was placed over a magnetic stirrer. Then, a certain amount of PEGDA, LiCl, and MXene was added into the SBMA/APS solution. The mixture was transferred into the PTFE mold after the mixture was completely dissolved. The size of the rectangular mold is 5 × 15 × 45 mm^3^, the bottom diameter of the cylindrical mold is 10 mm, and the height is 5 mm. Finally, the mold containing the mixed solution was placed in the oven at 60 °C for 1 h. The water contents in the as-prepared PSBMA, PSBMA-L, and PSBMA-LM hydrogels were calculated to be 34%, 42%, and 49%, respectively.

To verify the effect of PEGDA, hydrogels with different PEGDA contents were prepared using the same method according to the ratio in Table S3. In this work, the SBMA/APS/PEGDA hydrogel was denoted as PSBMA. The PSBMA hydrogels with different PEGDA contents were referred to as PSBMA-0.24%, PSBMA-0.48%, PSBMA-0.72%, PSBMA-0.96%, and PSBMA-1.20%, respectively. Then, to verify the effect of LiCl, hydrogels with different LiCl contents were prepared using the same method according to the ratio in Table S4. The SBMA/APS/PEGDA/LiCl hydrogel was denoted as PSBMA-L. The PSBMA-L hydrogels with different LiCl contents were referred to as PSBMA-L-2.5%, PSBMA-L-5%, PSBMA-L-7.5%, PSBMA-L-10%, and PSBMA-L-15%. Finally, to verify the effect of MXene, hydrogels with different MXene contents were prepared using the same method according to the ratio in Table S5. The SBMA/APS/PEGDA/LiCl/MXene hydrogel was denoted as PSBMA-LM. The PSBMA-LM hydrogels with different MXene contents were referred to as PSBMA-LM-0.5%, PSBMA-LM-1%, PSBMA-LM-1.5%, PSBMA-LM-2%, and PSBMA-LM-2.5%.

Preparation of the hydrogel strain sensor: The copper tape was attached on both sides of the hydrogel as an electrode, and the interface contact between the electrode and the hydrogel electrolyte is improved by using the adhesion of the hydrogel itself.

XRD analysis: The crystalline property of MXene was characterized by X-ray diffractometer (DY5261/Xpert3, CEM, Matthews, NC, USA) with the Cu Kα radiation (40 kV, 40 mA, λ = 0.1542 nm) at room temperature. The measurements were performed at the 2θ range of 5–80°.

SEM analysis: Morphological details of PSBMA-LM hydrogel and MXene were obtained by a field emission scanning microscope (FEI Nova Nano-SEM 230, FEI Company, Hillsboro, OR, USA) with the primary electron energy of 20.0 kV. To prepare suitable samples for measurement, the hydrogels were rapidly frozen with liquid nitrogen and carefully crushed. Subsequently, the fractured hydrogels were freeze-dried at −60 °C for 7 days to remove the frozen water. The freeze-dried hydrogels were then coated with gold and analyzed by scanning electron microscopy at an accelerating voltage.

XPS analysis: X-ray photoelectron spectroscopy (XPS) analysis was performed using an ESCALAB 250 X-ray photoelectron spectrometer (Thermo Fisher Scientific, Waltham, MA, USA) with a monochromated X-ray source (Al Kα hν = 1486.6 eV) under vacuum pressure less than 5.0 × 10^−7^ mBar, with a working voltage was 12 KV. The data were analyzed via XPSpeak 4.1 software.

Nuclear magnetic resonance spectroscopy: The reaction processes of PSBMA and PSBMA-L hydrogels were monitored by ^1^H nuclear magnetic resonance spectroscopy on a JEOL 400YH spectrometer (JEOL, Tokyo, Japan) using deuterated water (D_2_O) as the solvent. The monomer with or without LiCl was dissolved in D_2_O, then the samples were placed into NMR tubes, and detected at different reaction times.

FTIR analysis: Infrared spectroscopy was performed on a Nicolet 6700 FT-IR spectrometer (Thermo Fisher Scientific, Waltham, MA, USA) with KBr pellets in the 4000–500 cm^−1^ region.

Mechanical properties: The mechanical properties of the hydrogels were measured by a microcomputer-controlled mechanical property tester (CITEMA, Shanghai, China). In the tensile test, the hydrogels were cut into rectangles with dimensions of 30 × 10 × 1 mm^3^. The tensile rate was fixed at 1 mm/s.

Rheological measurement: Dynamic thermodynamic analysis (DMA/SDTA861e, Mettler Toledo, Columbus, OH, USA) was used to detect the G′ (storage modulus) and G″ (loss modulus) of the hydrogel gel formation process at a constant temperature of 25 °C with a frequency fluctuation (ω) of 10 rad s^−1^.

Conductive property: The ionic conductivity of the hydrogel was measured by the impedance spectrum in a frequency range of 1 to 1 × 10^6^ Hz using the electrochemical workstation (CHI 660E, Shanghai, China). The conductivity (σ) was calculated by the following formula:

σ=L/RS

where *L* (cm) was the distance between each two electrodes, *S*/cm^2^ was the cross-sectional area of hydrogels, and *R* (Ω) was the intercept through the horizontal axis in the EIS diagram.

Water retention property: PSBMA, PSBMA-L, and PSBMA-LM hydrogels were placed in the same environment for 7 days. The water retention capacity of the hydrogels at different times was calculated according to the following formula:

water retention (%) = m_t_/m_0_ × 100%

where m_0_ and m_t_ are the weights of the hydrogels on day 0 and day t, respectively.

## Data Availability

The original contributions presented in the study are included in the article/Supplementary Materials, further inquiries can be directed to the corresponding authors.

## Supplementary Materials

The following supporting information can be downloaded at: https://www.mdpi.com/article/10.3390/gels10110720/s1, Figure S1: The XRD curves of the MAX and MXene; Figure S2: SEM image of MXene nanosheets; Figure S3: Gelation process of the PSBMA, PSBMA-L, PSBMA-M, PSBMA-LM, and precursor solutions. Photographs indicate the appearance of the precursor solution after gelation; Figure S4: Gelation process of the PSBMA-M, PSBMA-LM, PSBMA-NM, PSBMA-KM, and precursor solutions. Photographs indicate the appearance of the precursor solution after gelation; Figure S5: PSBMA-LM hydrogel continuous 5 stretching cycle curve. (a) The cyclic strain is 15%. (b) The cyclic strain is 25%. (c) The cyclic strain is 40%. (d) The cyclic strain is 50%. (e) The cyclic strain is 65%. (f) The cyclic strain is 80%; Figure S6: Images of LiCl powder to absorb water at 25 °C and 54% RH; Figure S7: Weight ratio of PSBMA, PSBMA-L, and PSBMA-LM hydrogels during vacuum drying at 60 °C or regeneration at 25 °C and 54% RH for different periods; Figure S8: (a) Adhesion of PSBMA-LM hydrogel on different interfaces. (b) The adhesion of hydrogels to human skin; Figure S9: Tensile stress–strain curve of PSBMA-LM hydrogel for 10 min after self-healing; Figure S10: Relative resistance changes in the sensors for (a–c) small strain (6%, 9%, 30%) and (d-f) large strain (40%, 100%, 150%); Figure S11: Relative resistance variation curves of PSBMA-LM under different strains. Figure S12: (a–b) Real-time monitoring of wrist, elbow, and knee based on PSBMA-LM sensors. (d) Cyclic stability tests of PSBMA-LM hydrogel-based sensors under 100% strain; Figure S13: Schematic diagram of the flexible surface EMG acquisition and processing system; Figure S14: (a) Elbow motion characteristic amplitude signal. (b) Pulse motion characteristic amplitude signal. (c) Wrist motion characteristic amplitude signal; Figure S15: The characteristic signal diagram produced when the finger writes 26 capital letters; Figure S16: RandomForest model evaluation diagram; Figure S17: GradientBoosting model evaluation diagram; Figure S18: ExtraTrees model evaluation diagram; Figure S19: AdaBoost model evaluation diagram; Figure S20: DecisionTree model evaluation diagram; Figure S21: KNeighbors model evaluation diagram; Table S1: Main properties of MXene based hydrogel flexible sensor [32,33,34,35,36,37,38,39,40]; Table S2: Accuracy of different algorithm models; Table S3: The experimental ingredients and nomenclatures of the PSBMA-L hydrogel; Table S4: The experimental ingredients and nomenclatures of the PSBMA-LM hydrogel; Table S5: The experimental ingredients and nomenclatures of the PSBMA-LM hydrogel.; Video S1: The measurement of mechanical properties.
