# Supplementary material for: Alkali Ion-Accelerated Gelation of MXene-Based Conductive Hydrogel for Flexible Sensing and Machine Learning-Assisted Recognition
Source: Gels. 2024 Nov 7;10(11):720. doi: 10.3390/gels10110720 (PMC11593876; doi:10.3390/gels10110720)
Supplement: Supplementary file 1 [file gels-10-00720-s001.zip › gels-3286368-supplementary.pdf]

gels

MDPI

Supporting Information

Article

Alkali Ion-Accelerated Gelation of MXene-Based Conductive Hydrogel for Flexible Sensing and Machine Learning-Assisted Recognition

Weidan Na [1,†], Chao Xu [2,†], Lei An [2], Changjin Ou [2,*], Fan Gao [2], Guoyin Zhu [2] and Yizhou Zhang [2,*]

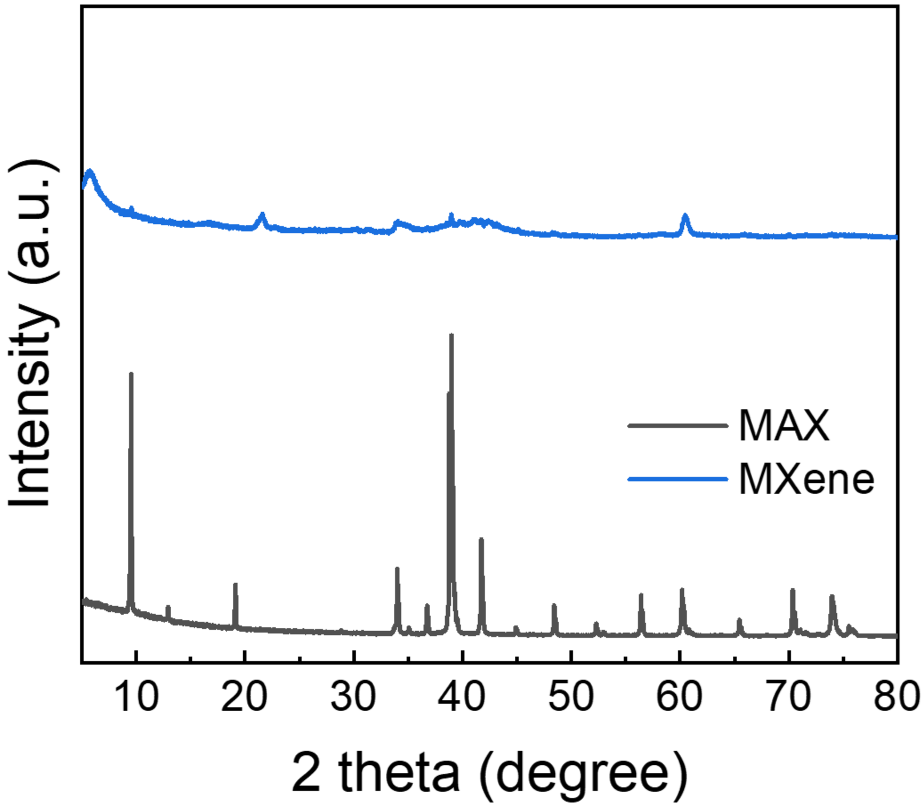

Figure S1. The XRD curves of the MAX and MXene.

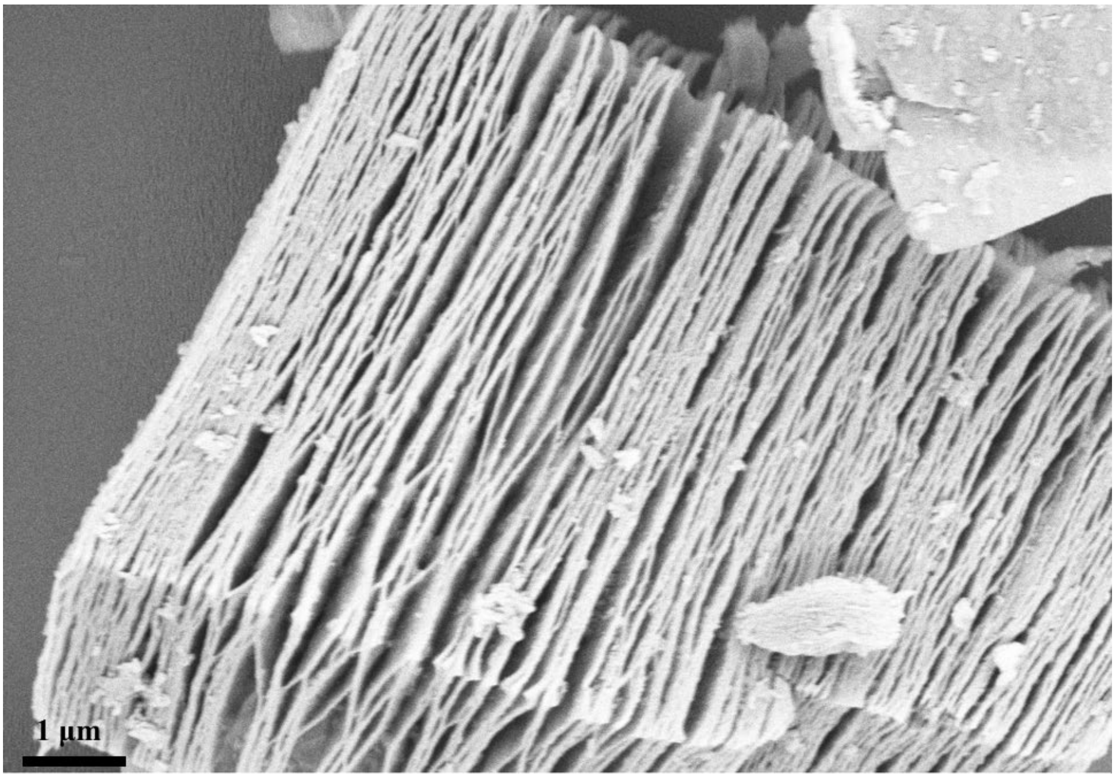

Figure S2. SEM image of MXene nanosheets.

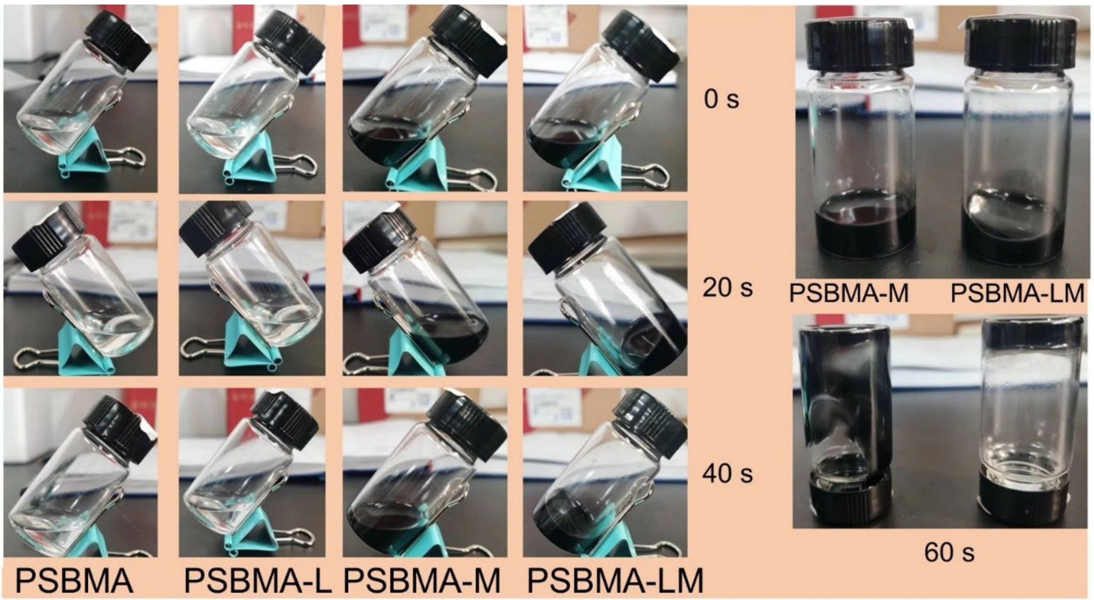

Figure S3. Gelation process of the PSBMA, PSBMA-L, PSBMA-M, PSBMA-LM, precursor solutions. Photographs indicate the appearance of the precursor solution after gelation.

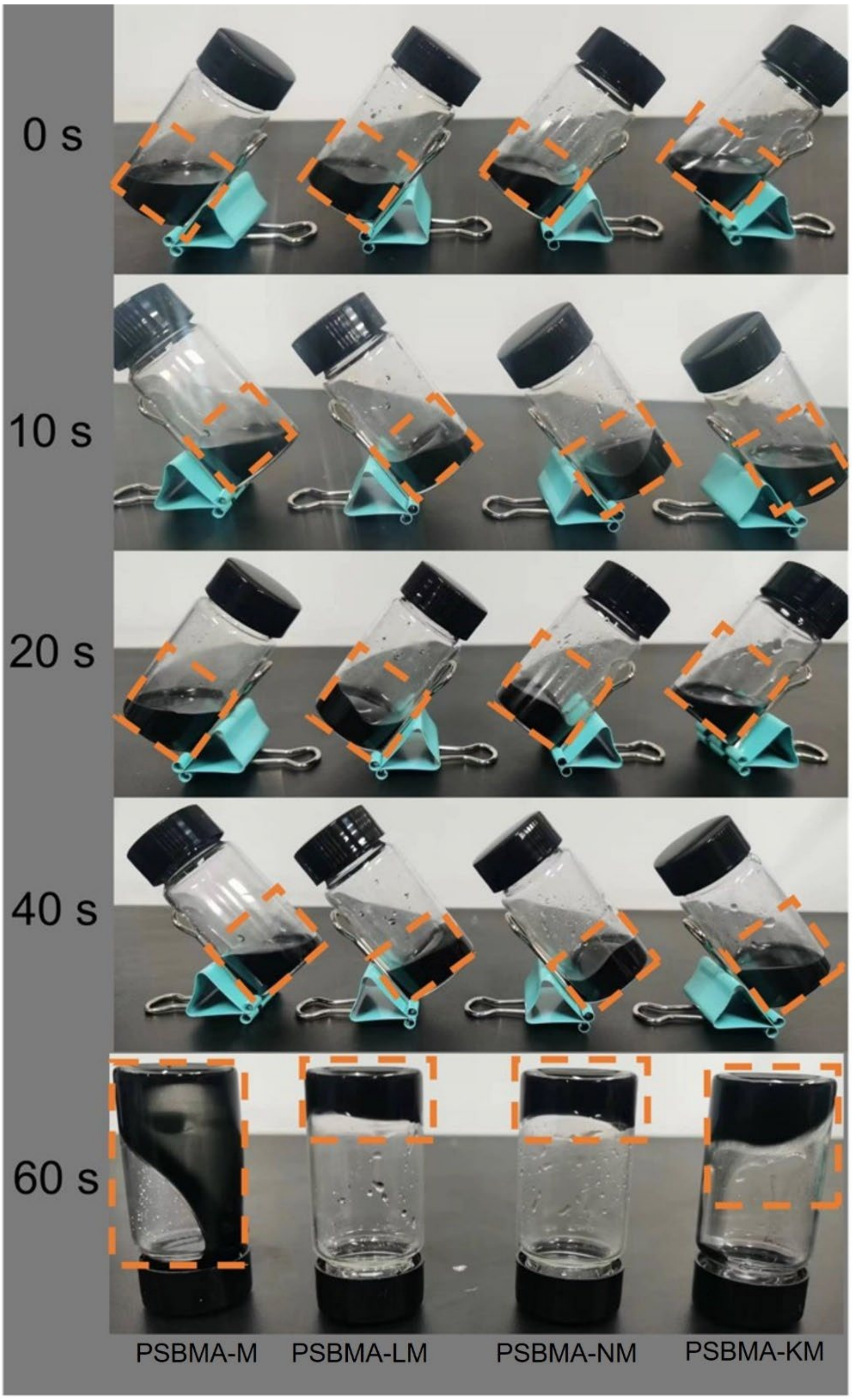

Figure S4. Gelation process of the PSBMA-M, PSBMA-LM, PSBMA-NM, PSBMA-KM, precursor solutions. Photographs indicate the appearance of the precursor solution after gelation.

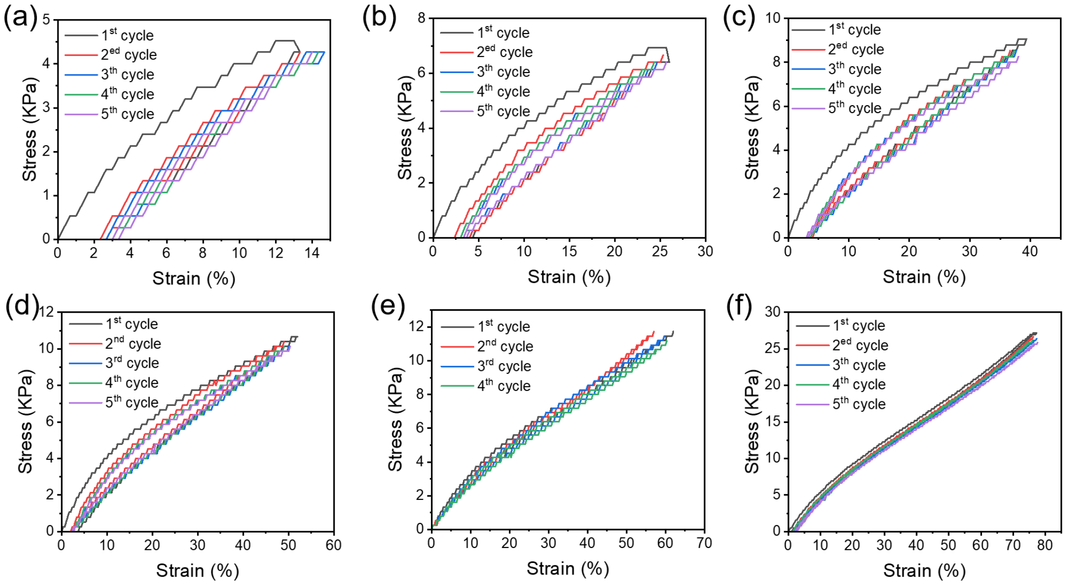

Figure S5. PSBMA-LM hydrogel continuous 5 stretching cycle curve. (a) The cyclic strain is 15%. (b) The cyclic strain is 25%. (c) The cyclic strain is 40%. (d) The cyclic strain is 50%. (e) The cyclic strain is 65%. (f) The cyclic strain is 80%.

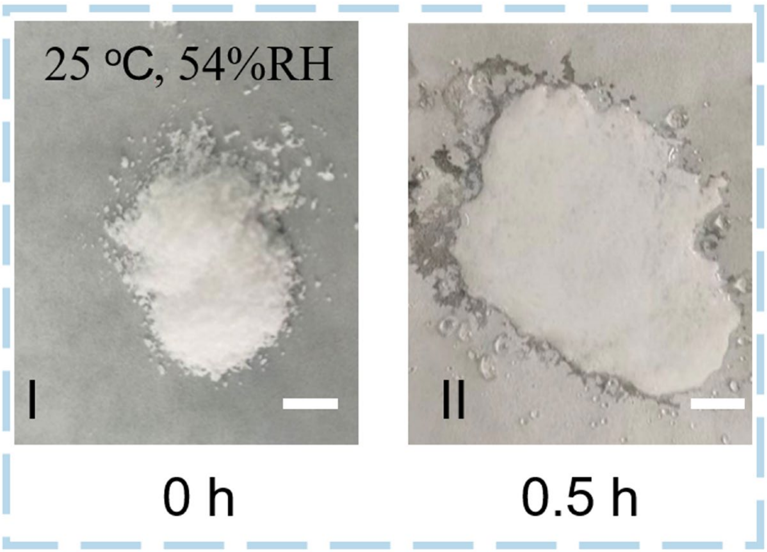

Figure S6. Images of LiCl powder to absorb water at 25 °C and 54% RH. The scale bar is ~2.0 mm.

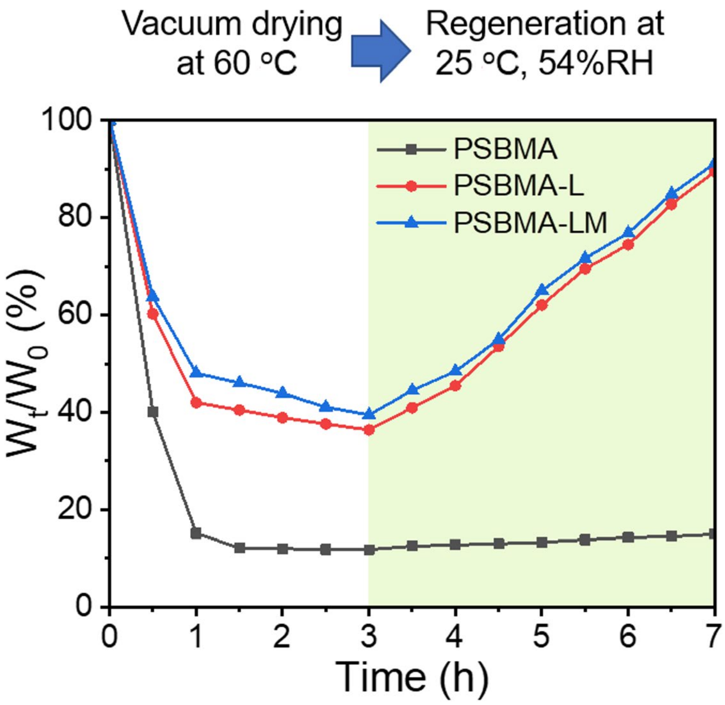

Figure S7. Weight ratio of PSBMA, PSBMA-L, PSBMA-LM hydrogels during vacuum drying at 60 °C or regeneration at 25 °C and 54% RH for different periods.

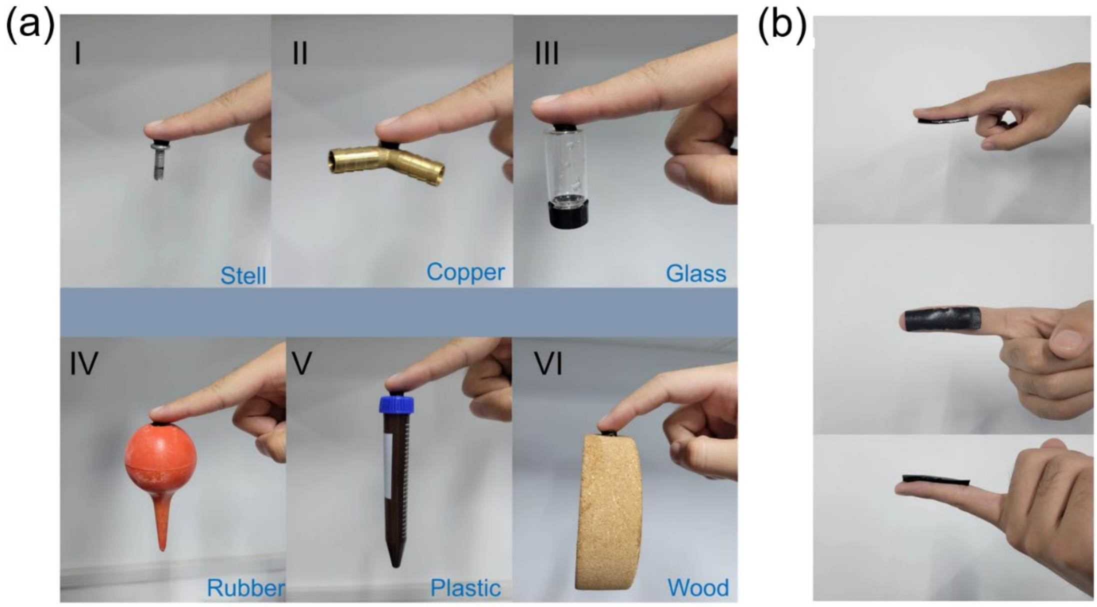

Figure S8. (a) Adhesion of PSBMA-LM hydrogel on different interfaces. (b) The adhesion of hydrogels to human skin.

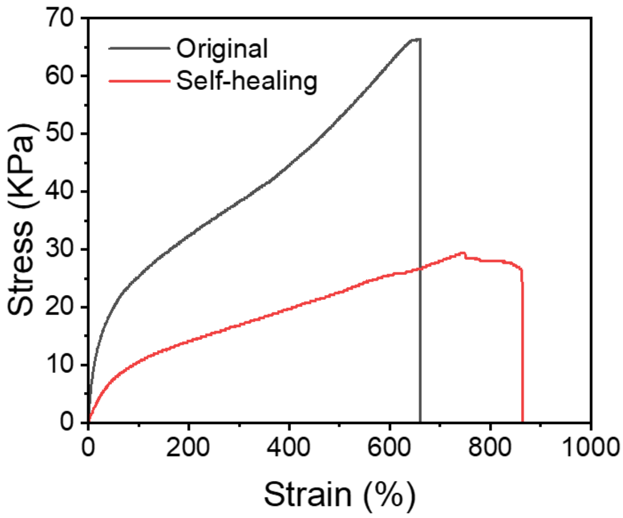

Figure S9. Tensile stress-strain curve of PSBMA-LM hydrogel for 10 minutes after self-healing.

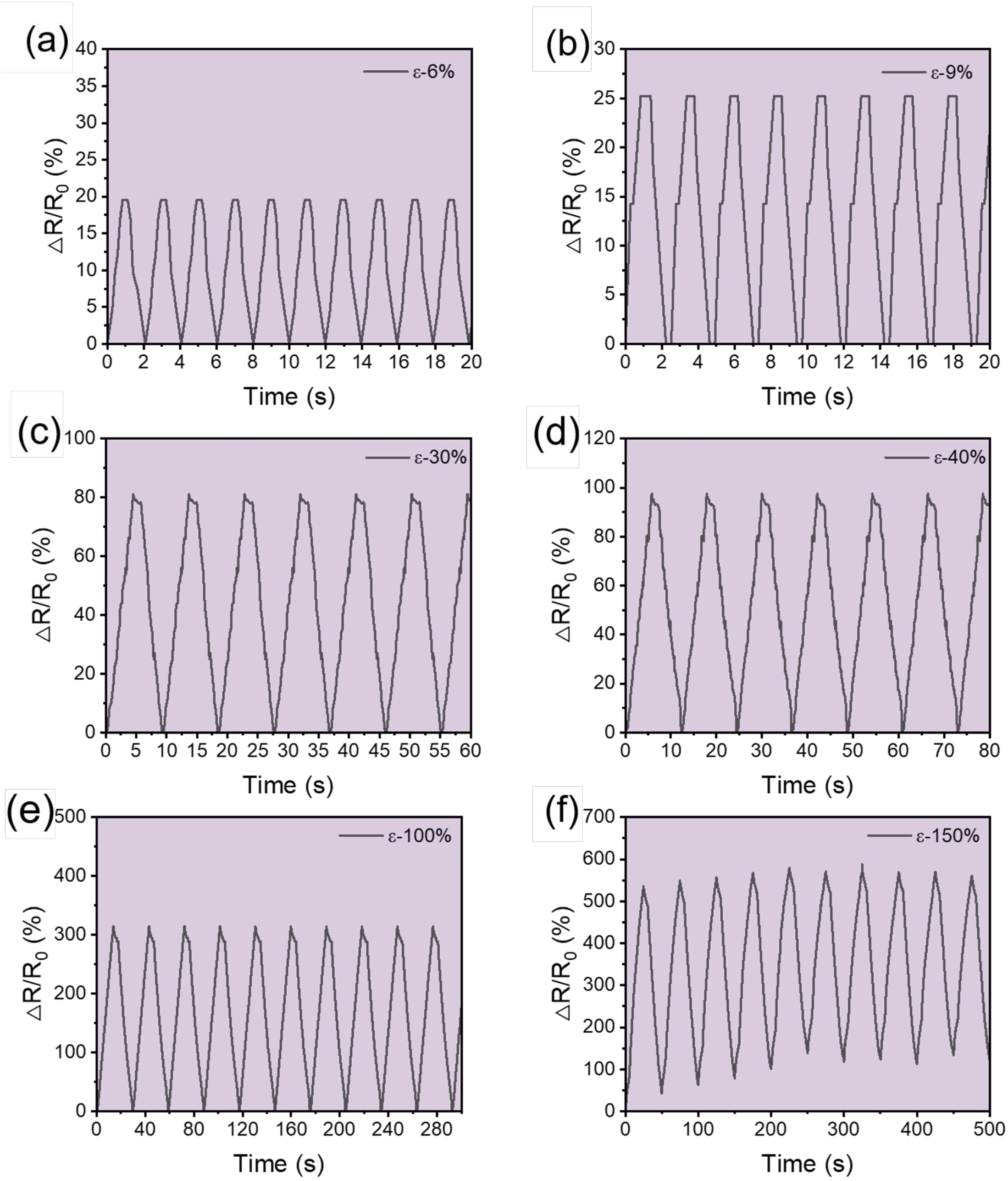

Figure S10. Relative resistance changes in the sensors for (a-c) small strain (6%, 9%, 30%) and (d-f) large strain (40%, 100%, 150%).

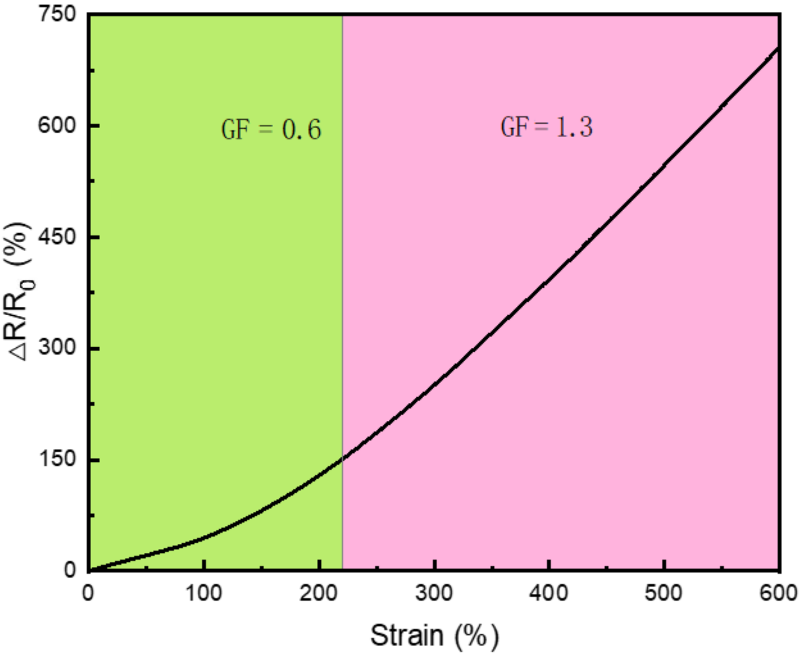

Figure S11. Relative resistance variation curve of PSBMA-LM under different strains. The gauge factor (GF) is 0.6 and 1.3 in the strain range of 0-220% and 220-600%, respectively.

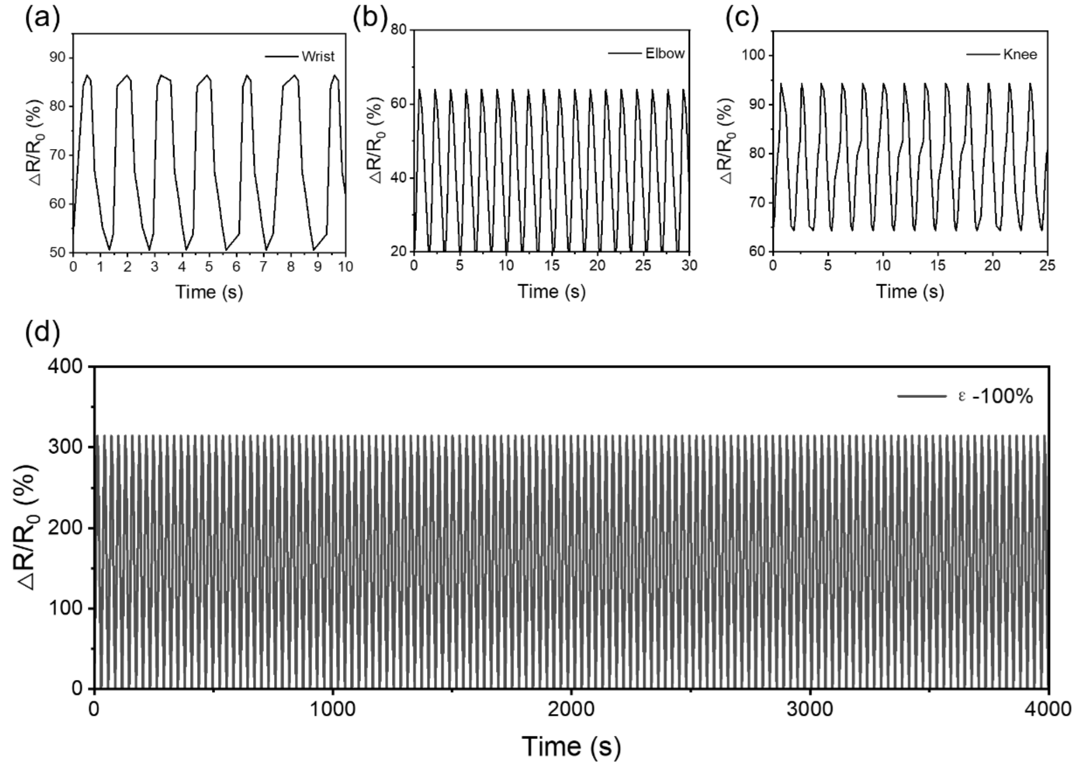

Figure S12. (a-b) Teal-time monitoring of wrist, elbow and knee based on PSBMA-LM sensors. (d) Cyclic stability tests of PSBMA-LM hydrogel-based sensor under 100% strain.

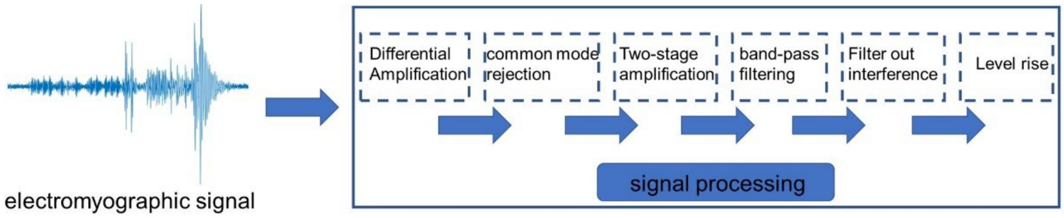

Figure S13. Schematic diagram of flexible surface EMG acquisition and processing system.

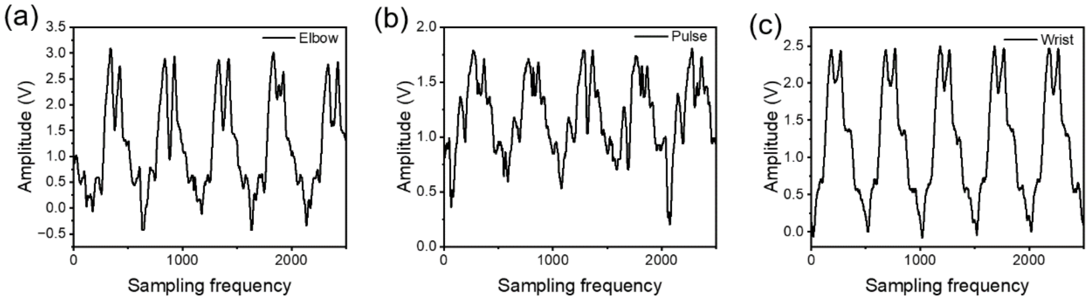

Figure S14. (a) Elbow motion characteristic amplitude signal. (b) Pulse motion characteristic amplitude signal. (c) Wrist motion characteristic amplitude signal.

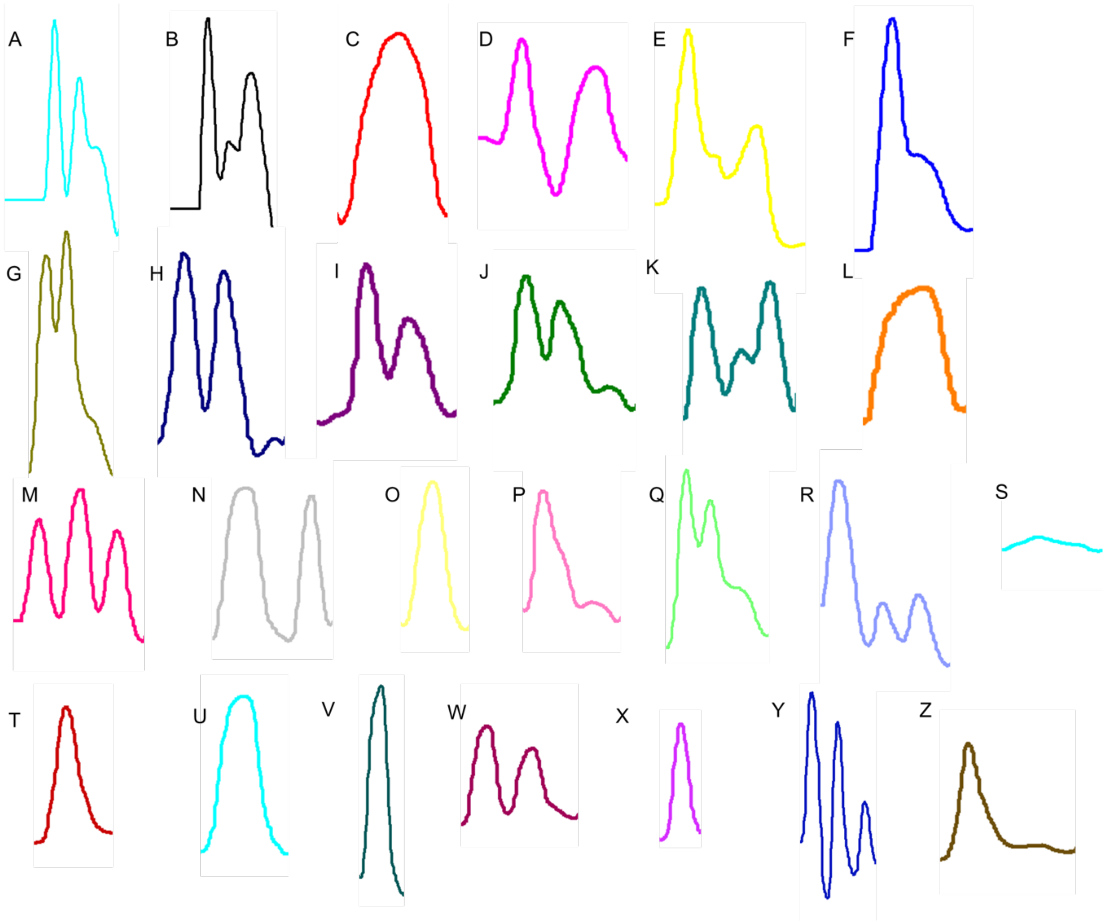

Figure S15. The characteristic signal diagram produced when the finger writes 26 capital letters.

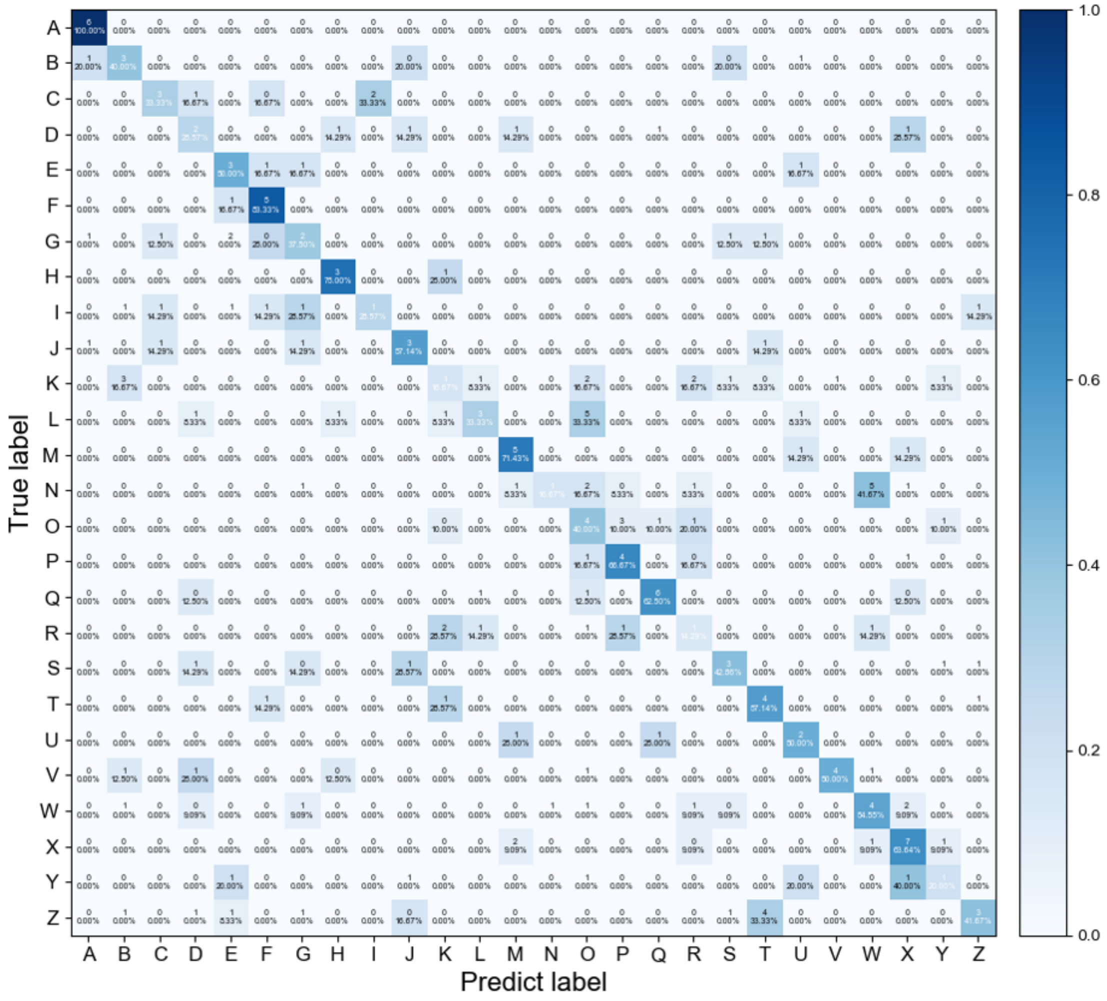

Figure S16. RandomForest model evaluation diagram.

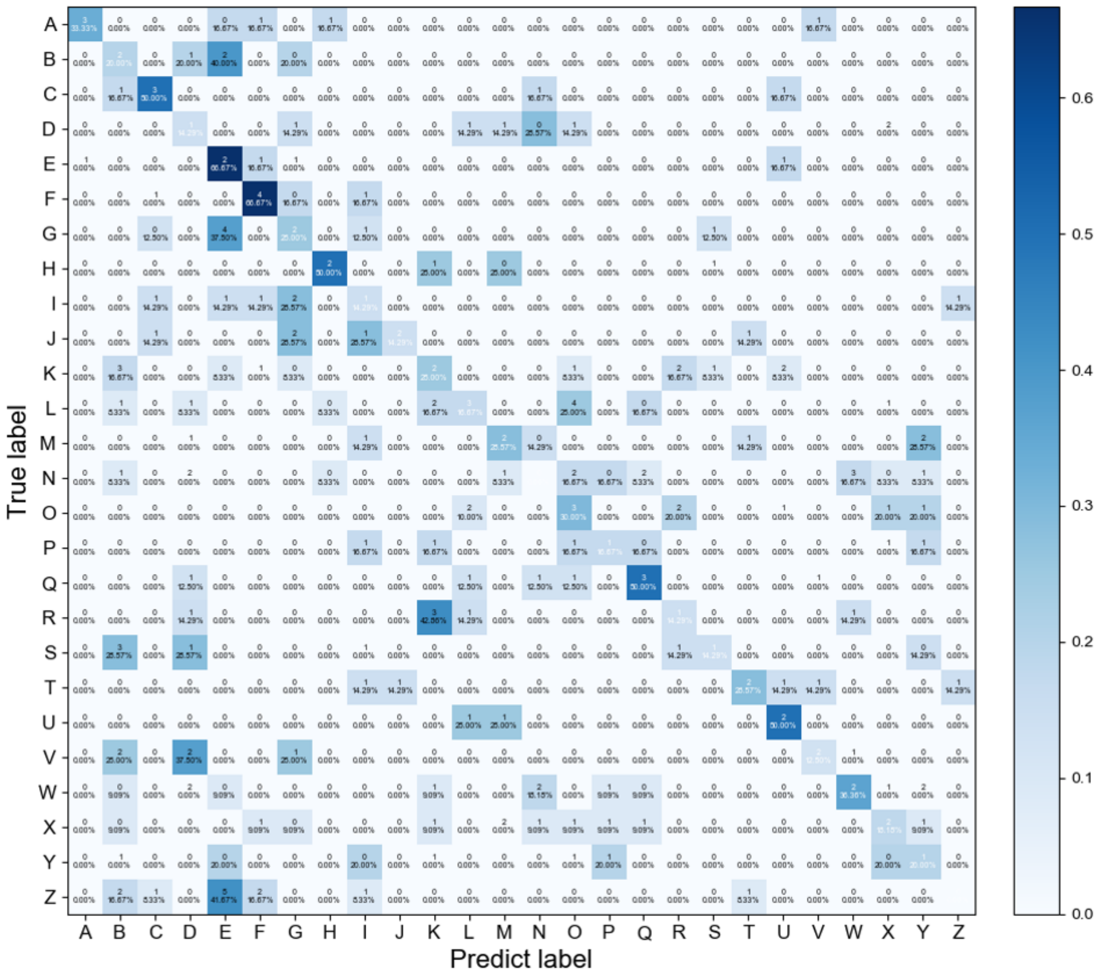

Figure S17. GradientBoosting model evaluation diagram.

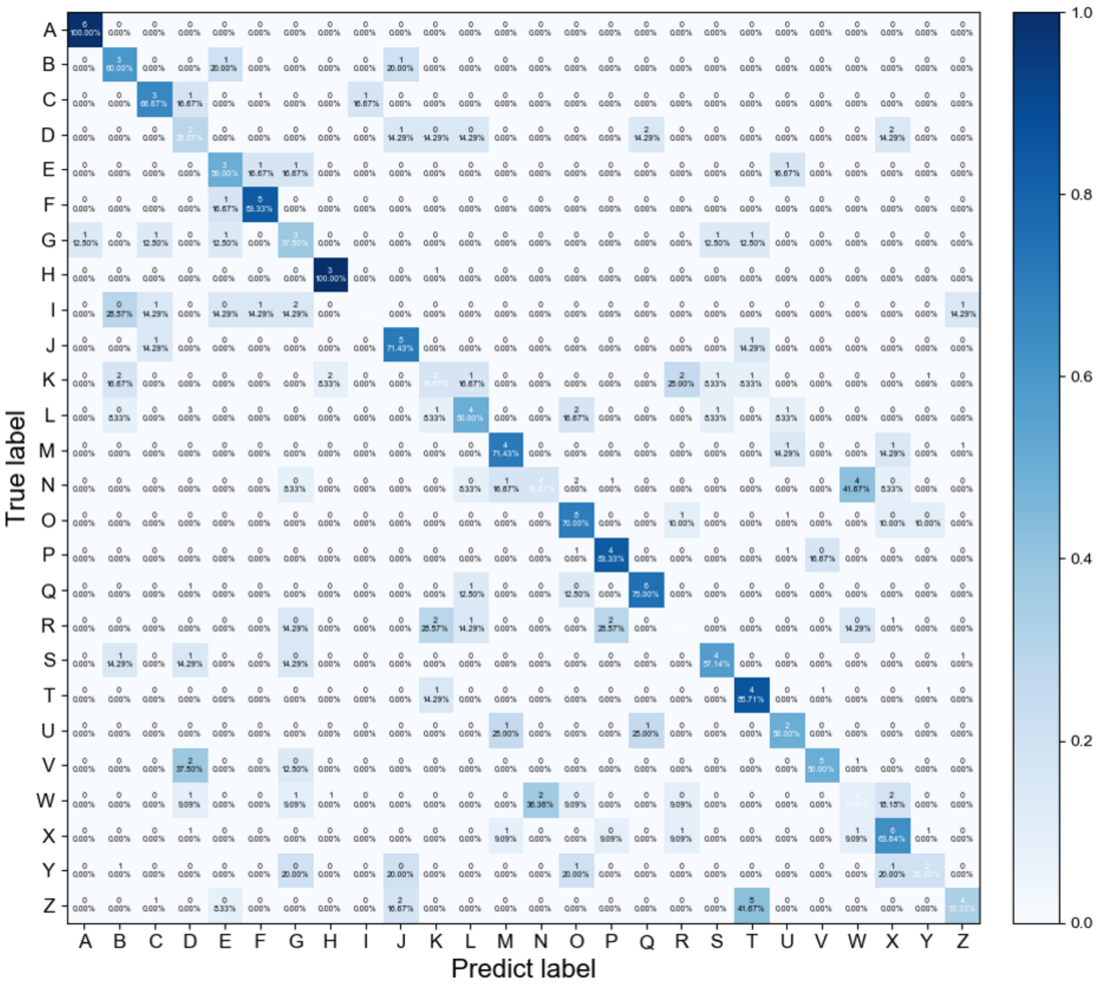

Figure S18. ExtraTrees model evaluation diagram.

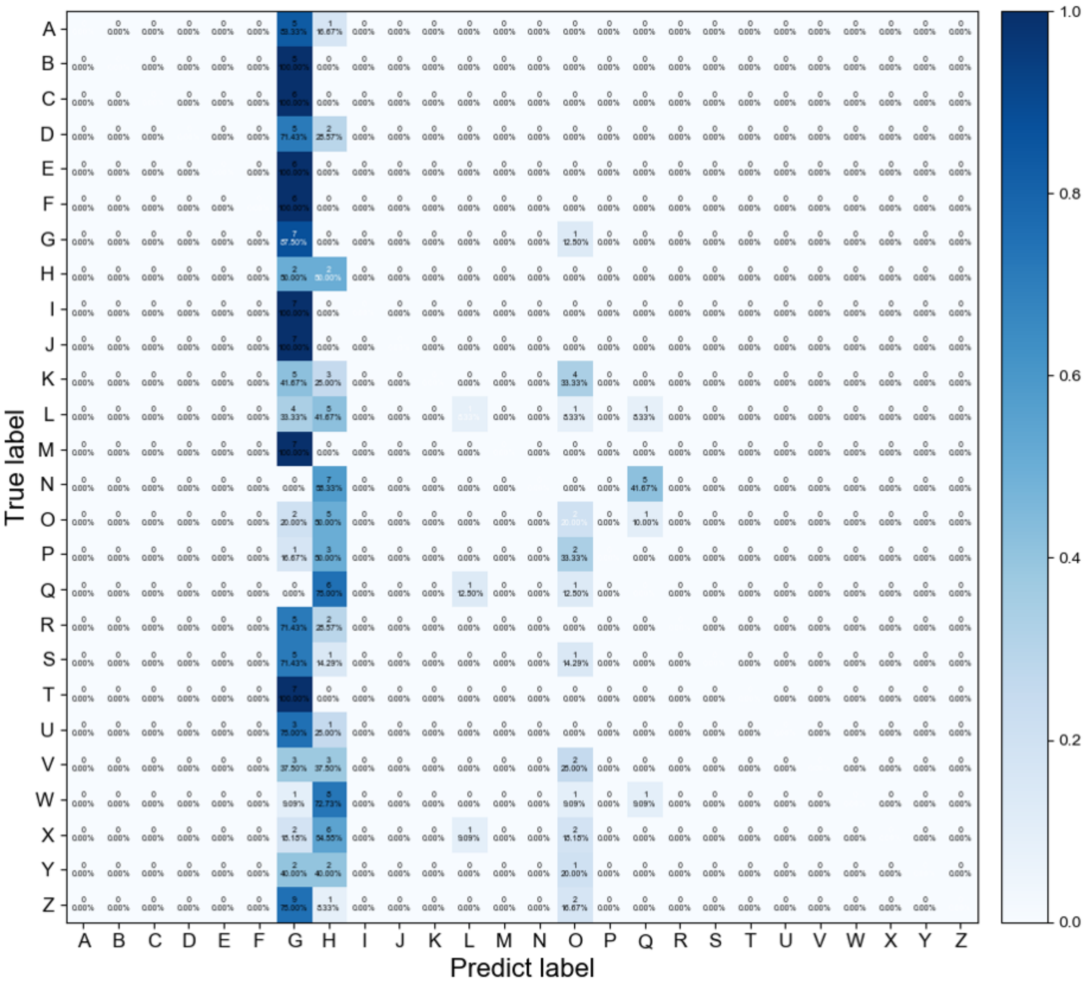

Figure S19. AdaBoost model evaluation diagram.

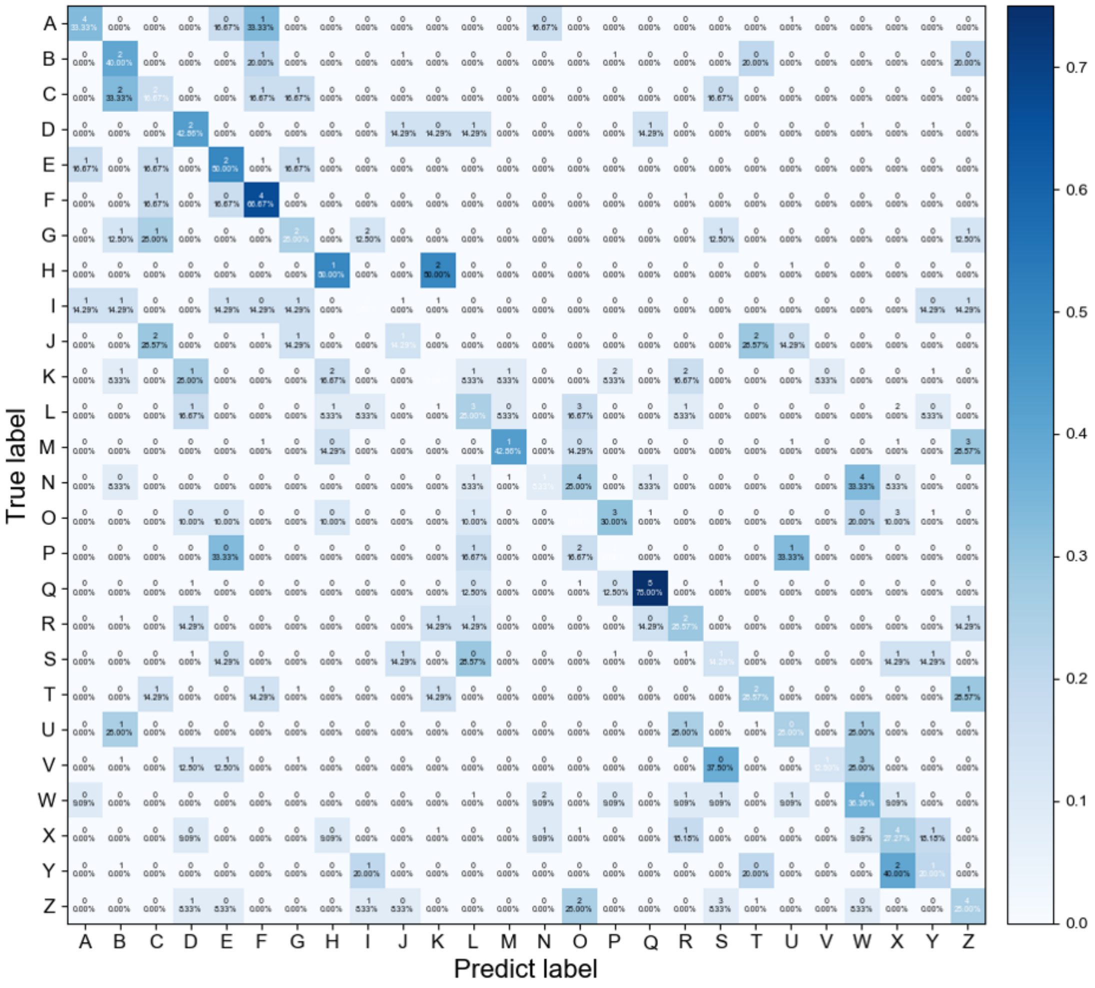

Figure S20. DecisionTree model evaluation diagram.

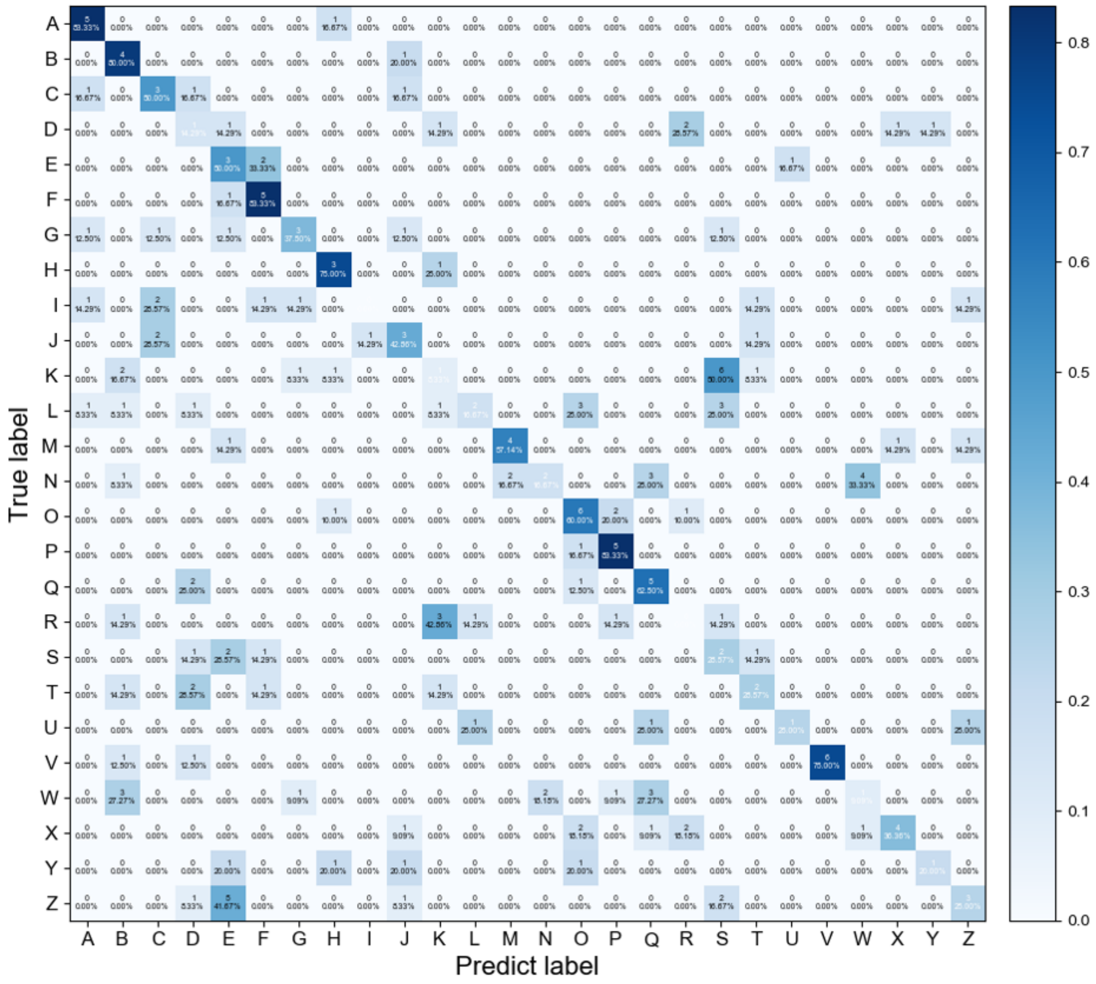

Figure S21. KNeighbors model evaluation diagram.

Table S1. Main properties of MXene based hydrogel flexible sensor.

Materials	Max strain (%)	Conductivity (S/cm)	Minimum temperature	GF	Ref.
MXene/SBMA/LiCl	1090	0.12	-20	1.3	This work
MXene/PAM/Agar	1300	0.0102	-26	2.99（0-200%）	[1]
MXene/PVA/$ZnSO_4$	247	0.00056	-36	3.42(0-50%)	[2]
MXene/ Polyacrylamide -co-Acrylic acid / Chitosan	1000	0.0134	-20	/	[3]
MXene-PAA-Fe^{3+}	3080	0.038	/	10.09	[4]
MXene/ Sodium carboxymethyl cellulose /Polyacrylic acid	1127	0.0109	/	5.79 (0-700%)	[5]
MXene/TEMPO/Bacterial cellulose	2800	/	-20	15.65	[6]
MXene/Agar/ Borax	105.1	0.0814	/	1.52	[7]
PVA/PEG/TA-MXene-Na^+ /Li^+	400	0.081	-27	1.12	[8]
MXene/PEDOT:PSS	560	0.1176	/	9.93	[9]

Table S2. Accuracy of different algorithm models.

Classifier	Accuracy
RandomForest	0.42
GradientBoosting	0.24
ExtraTree	0.57
AdaBoost	0.06
DecisionTree	0.26
KNeighbors	0.37

Table S3. The experimental ingredients and nomenclatures of the PSBMA hydrogel.

Sample	SBMA (g)	APS (g)	PEGDA (mg)	LiCl (g)	MXene (g)	Water (g)
PSBMA-0.24%	2	0.02	4.8	0	0	3
PSBMA-0.48%	2	0.02	9.6	0	0	3
PSBMA-0.72%	2	0.02	14.4	0	0	3
PSBMA-0.96%	2	0.02	19.2	0	0	3
PSBMA-1.2%	2	0.02	24	0	0	3

Table S4. The experimental ingredients and nomenclatures of the PSBMA-L hydrogel.

Sample	SBMA (g)	APS (g)	PEGDA (mg)	LiCl (g)	Mxene (g)	Water (g)
PSBMA-L-2.5%	2	0.02	14.4	0.05	0	3
PSBMA-L-5%	2	0.02	14.4	0.1	0	3
PSBMA-L-7.5%	2	0.02	14.4	0.15	0	3
PSBMA-L-10%	2	0.02	14.4	0.2	0	3
PSBMA-L-15%	2	0.02	14.4	0.3	0	3

Table S5. The experimental ingredients and nomenclatures of the PSBMA-LM hydrogel.

Sample	SBMA (g)	APS (g)	PEGDA (mg)	LiCl (g)	Mxene (g)	Water (g)
PSBMA-LM-0.5%	2	0.02	14.4	0.2	0.01	3
PSBMA-LM-1%	2	0.02	14.4	0.2	0.02	3
PSBMA-LM-1.5%	2	0.02	14.4	0.2	0.03	3
PSBMA-LM-2%	2	0.02	14.4	0.2	0.04	3
PSBMA-LM-2.5%	2	0.02	14.4	0.2	0.05	3

Video 1. The measurement of mechanical properties.

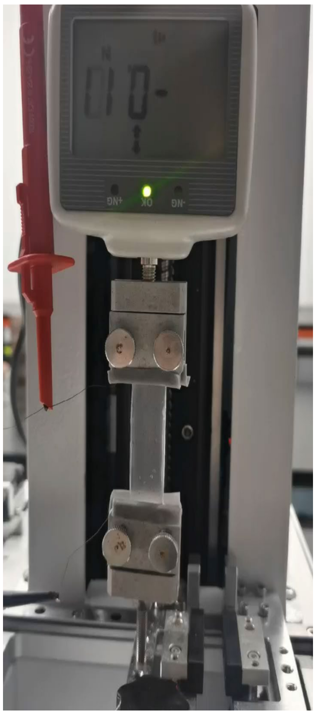

References

32. Chen, K.; Lai, W.; Xiao, W.; Li, L.; Huang, S.; Xiao, X. Low-Temperature Adaptive Dual-Network MXene Nanocomposite Hydrogel as Flexible Wearable Strain Sensors. *Micromachines* **2023**, *14*, 1563.
33. Feng, Y.; Liu, H.; Zhu, W.; Guan, L.; Yang, X.; Zvyagin, A.V.; Zhao, Y.; Shen, C.; Yang, B.; Lin, Q. Muscle-inspired MXene conductive hydrogels with anisotropy and low-temperature tolerance for wearable flexible sensors and arrays. *Adv. Funct. Mater.* **2021**, *31*, 2105264.
34. Li, S.-N.; Yu, Z.-R.; Guo, B.-F.; Guo, K.-Y.; Li, Y.; Gong, L.-X.; Zhao, L.; Bae, J.; Tang, L.-C. Environmentally stable, mechanically flexible, self-adhesive, and electrically conductive $Ti_3C_2T_X$ MXene hydrogels for wide-temperature strain sensing. *Nano Energy* **2021**, *90*, 106502.
35. Li, Y.; Yan, J.; Liu, Y.; Xie, X.M. Super Tough and Intelligent Multibond Network Physical Hydrogels Facilitated by $Ti_3C_2T_x$ MXene Nanosheets. *ACS Nano* **2022**, *16*, 1567.
36. Yin, H.; Liu, F.; Abdiryim, T.; Chen, J.; Liu, X. Sodium carboxymethyl cellulose and MXene reinforced multifunctional conductive hydrogels for multimodal sensors and flexible supercapacitors. *Carbohydr. Polym.* **2024**, *327*, 121677.
37. Dong, B.; Yu, D.; Lu, P.; Song, Z.; Chen, W.; Zhang, F.; Li, B.; Wang, H.; Liu, W. TEMPO bacterial cellulose and MXene nanosheets synergistically promote tough hydrogels for intelligent wearable human-machine interaction. *Carbohydr. Polym.* **2024**, *326*, 121621.
38. Nie, Z.; Peng, K.; Lin, L.; Yang, J.; Cheng, Z.; Gan, Q.; Chen, Y.; Feng, C. A conductive hydrogel based on nature polymer agar with self-healing ability and stretchability for flexible sensors. *Chem. Eng. J.* **2023**, *454*, 139843.
39. Yuan, S.; Bai, J.; Li, S.; Ma, N.; Deng, S.; Zhu, H.; Li, T.; Zhang, T. A multifunctional and selective ionic flexible sensor with high environmental suitability for tactile perception. *Adv. Funct. Mater.* **2023**, *34*, 2309626.
40. Xue, P.; Valenzuela, C.; Ma, S.; Zhang, X.; Ma, J.; Chen, Y.; Xu, X.; Wang, L. Highly conductive MXene/PEDOT: PSS-integrated poly (N-Isopropylacrylamide) hydrogels for bioinspired somatosensory soft actuators. *Adv. Funct. Mater.* **2023**, *33*, 2214867.
